# The gut–disc axis in intervertebral disc degeneration: microbial metabolites, immunometabolic remodeling, and regulated cell death

**DOI:** 10.3389/fcimb.2026.1960350

**Published:** 2026-10-29

**Authors:** HaoYang Wu, DeChao Yuan, RenJian He, ShiWei Liu

**Affiliations:** Department of Orthopedics, Zigong First Peoples Hospital, Zigong, China

**Keywords:** extracellular vesicles, ferroptosis, gut microbiota, gut–disc axis, immunometabolism, intervertebral disc degeneration, microbial metabolites, NLRP3 inflammasome

## Abstract

Intervertebral disc degeneration (IDD) involves interactions among mechanical injury, inflammation, and metabolic dysfunction. The proposed gut–disc axis extends this framework to intestinal microbial signals, but the strength and tissue relevance of the supporting evidence vary considerably. This narrative review examines whether microbial metabolites could influence IDD through pyroptosis, a gasdermin-mediated form of lytic regulated cell death, and ferroptosis, an iron-dependent form of regulated cell death driven by lipid peroxidation. We distinguish human microbial associations, experimental findings in disc tissues, mechanisms demonstrated in other organs, and computational predictions. Selected microbial metabolites regulate inflammatory and antioxidant pathways in non-disc models, whereas disc studies establish the relevance of mitochondrial dysfunction, iron handling, and local metabolite-dependent protein modifications. These findings support mechanistic hypotheses but do not establish that defined gut-derived metabolites reach the disc and causally regulate either death program. We also assess microbiome-directed interventions and local therapeutic strategies, emphasizing that efficacy against a downstream disc pathway does not demonstrate a microbial initiating mechanism. Clinical intervention evidence remains limited and does not establish structural disease modification. Translation requires verified metabolite exposure, cell-specific target engagement, functional validation of cell death, and longitudinal assessment of imaging, pain, and disability. This evidence-based distinction provides a framework for prioritizing testable gut–disc mechanisms and clinically relevant interventions.

## 1 Introduction

Low back pain is a major cause of disability worldwide (Global, regional, and national burden of low back pain, 1990-2020, its attributable risk factors, and projections to 2050: a systematic analysis of the Global Burden of Disease Study 2021, 2023). Intervertebral disc degeneration (IDD) can contribute to pain, although degenerative imaging findings also occur in asymptomatic individuals (Brinjikji et al., 2015). The intervertebral disc (IVD) is largely avascular, and its homeostasis depends on the interaction of matrix integrity, nutrient transport, and cellular metabolism. Degeneration disrupts these processes and is accompanied by inflammatory and catabolic signaling (Bermudez-Lekerika et al., 2022). Understanding systemic influences on this local pathology may reveal additional therapeutic targets.

The intestinal microbiota can substantially influence circulating metabolite profiles, providing a biological basis for communication with distant tissues (Wikoff et al., 2009). Within the proposed gut–disc framework, altered intestinal permeability, microbial products, immune regulation, and metabolite signaling represent potential routes of communication (Li et al., 2022). Bacterial extracellular vesicles provide an additional route supported by recent preclinical IDD experiments (Guan et al., 2026). A pilot human case–control study identified distinct gut microbial profiles in patients with disc degeneration, including depletion of several short-chain fatty acid-producing taxa and associations with circulating inflammatory markers and Modic changes (Rajasekaran et al., 2024). A subsequent study integrating fecal 16S rDNA sequencing with untargeted metabolomics reported altered abundances of Proteobacteria and Fusobacteriota and enrichment of lipid-related metabolites in patients with IDD (Liu J. et al., 2025). Mendelian randomization has further suggested potential causal associations between selected microbial taxa and intervertebral disc disorders, although such genetic evidence does not identify the responsible microbial functions, circulating mediators, or target cells within the disc (Ge et al., 2025).

Recent mechanistic studies have begun to move the field beyond association. In particular, reduced fecal abundance of Akkermansia muciniphila was associated with greater IDD severity, whereas administration of A. muciniphila, its extracellular vesicles, or the vesicle-enriched effector protein B2UKX5 attenuated degeneration in several experimental models (Guan et al., 2026). These findings support the biological plausibility of long-range microbial communication with the disc. Nevertheless, evidence for sustained bacterial colonization of the IVD remains uncertain. Disc microbiome findings have shown limited reproducibility, and low microbial biomass makes these samples particularly susceptible to contamination during collection and laboratory processing (Mengis et al., 2024; Fierer et al., 2025). The gut–disc axis should therefore not be reduced to direct bacterial migration. At present, circulating microbial products, bacterial extracellular vesicles, immune-cell reprogramming, and microbiota-derived metabolites provide more defensible mechanisms of gut-to-disc communication.

Microbial metabolites are attractive candidate mediators because microbiota-dependent metabolites can be detected in the circulation (Wikoff et al., 2009). Studies in non-disc tissues have further demonstrated metabolite-dependent regulation of macrophage receptor signaling, inflammatory responses, and ferroptosis-related antioxidant defenses (Dong et al., 2025; Hua et al., 2025; Luo et al., 2025). Whether comparable effects occur within the IVD requires direct investigation. Network pharmacology and molecular docking have nominated acetate, propionate, butyrate, succinate, trimethylamine N-oxide, and indole-3-propionic acid as candidate regulators of IDD-associated inflammatory and metabolic targets, including IL-6, TLR4, CXCL8, and JUN (Liu et al., 2026b). However, such computational associations do not establish metabolite production by a specific microbial strain, systemic bioavailability, entry into the disc, or target engagement in disc cells. A rigorous gut–disc model must distinguish demonstrated microbial contributions from local host metabolism. Disc cells function in a nutrient- and diffusion-constrained environment, and recent IDD studies have implicated local lactate metabolism in oxidative injury and inflammatory responses (Horner and Urban, 2001; Xia et al., 2026; Zhang Y. et al., 2026). Intradiscal lactate detection alone therefore does not establish a gut microbial origin.

Pyroptosis and ferroptosis represent two complementary forms of regulated cell death through which microbial and metabolic signals may be translated into progressive disc injury. Pyroptosis is a form of regulated cell death executed by gasdermin-mediated membrane permeabilization; inflammatory caspase-dependent cleavage of gasdermin D (GSDMD) provides a central mechanism linking inflammatory signaling to this process (Dixon et al., 2012; Shi et al., 2015). Ferroptosis is an iron-dependent form of regulated cell death characterized by lipid peroxide accumulation, with glutathione peroxidase 4 (GPX4) acting as a key protective regulator (Dixon et al., 2012; Yang et al., 2014). Oxidative stress-induced ferroptosis has been demonstrated in both nucleus pulposus and annulus fibrosus cells, supporting its relevance across multiple disc compartments (Yang et al., 2021). Separate IDD studies implicate mitochondrial dysfunction and impaired mitophagy in pyroptosis (Ma et al., 2022), and dysfunction of nuclear factor erythroid 2-related factor 2 (NRF2), ferritinophagy, and inflammatory oxidative stress in ferroptosis (Ma et al., 2024). These findings suggest potentially shared regulatory processes, but do not establish that both death programs are activated by the same mechanism in the same cell population. Microbial metabolites may therefore function not as simple protective or harmful molecules, but as context-dependent regulators of a shared immune–metabolic network.

Despite this conceptual appeal, several critical gaps remain. The studies reviewed here provide limited direct information on microbial-metabolite concentrations within human discs. Endplate transport constraints also mean that circulating concentrations cannot be assumed to represent intradiscal exposure (Ren et al., 2023). Metabolite effects are context-dependent, as illustrated by the opposing ferroptosis-related effects of butyrate in inflammatory and cancer models (Liu L. et al., 2025; Chen et al., 2026). Moreover, changes in NLR family pyrin domain-containing 3 (NLRP3), GPX4, acyl-CoA synthetase long-chain family member 4 (ACSL4), or oxidative-stress markers alone do not establish execution of pyroptosis or ferroptosis; pathway-specific functional validation is required (Mishima et al., 2025; Otchere et al., 2025). Causal attribution requires pathway-specific genetic or pharmacological rescue and, ideally, simultaneous measurement of microbial function, metabolite exposure, cellular target engagement, regulated cell death, and structural disease progression.

Accordingly, this review evaluates whether microbial metabolites can be causally linked to pyroptosis and ferroptosis in IDD. It distinguishes human associations, disc-specific experimental findings, non-disc mechanisms, and computational predictions, with particular attention to intradiscal exposure and the requirements for clinical translation.

## 2 Anatomical and immunological basis of the gut–disc axis

### 2.1 Structural organization, avascularity, and transport constraints

The IVD is a composite fibrocartilaginous organ composed of the central nucleus pulposus (NP), the surrounding annulus fibrosus (AF), and the superior and inferior cartilage endplates (CEPs). These compartments have distinct compositions and functions but operate as an integrated mechanical and metabolic unit (Sun et al., 2020). The NP contains a highly hydrated extracellular matrix enriched in aggrecan and type II collagen. Negatively charged glycosaminoglycan chains associated with aggrecan retain water and generate osmotic swelling pressure, allowing the NP to resist compression and distribute axial loads. The AF comprises concentric collagen-rich lamellae with alternating fiber orientations. This anisotropic organization constrains radial expansion of the pressurized NP and provides resistance to tensile, torsional, shear, and bending forces. The CEPs form an important interface for molecular transport between the vertebral compartment and the largely avascular inner disc, and degenerative changes in the CEP can reduce solute diffusivity (Crump et al., 2023; Ren et al., 2023).

The mature IVD is the largest avascular organ in the human body. Blood vessels are largely confined to the outer AF, vertebral marrow, and endplate-associated vascular channels, whereas the inner AF and NP lack direct vascular and lymphatic supply (Sun et al., 2020). Oxygen, glucose, and other small solutes must therefore diffuse from vertebral capillaries across the CEP and through the dense extracellular matrix. Metabolic waste products, including lactate, leave the disc through the same diffusion-dependent routes. This transport architecture creates a physiologically hypoxic, nutrient-limited, acidic, and hyperosmotic niche. Disc cells are adapted to these conditions, but their viability and matrix-synthetic capacity remain highly sensitive to further reductions in nutrient availability or waste clearance (Bermudez-Lekerika et al., 2022).

Avascularity contributes to relative immune sequestration of the NP by limiting direct vascular and immune-cell access (Sun et al., 2020). It also imposes transport constraints that must be considered when evaluating nutrient supply and the potential delivery of systemic therapeutics (Crump et al., 2023; Ren et al., 2023). CEP calcification and degeneration can reduce solute diffusivity across the disc–vertebral interface, thereby compromising nutrient supply and waste-product clearance within the avascular disc (Crump et al., 2023; Ren et al., 2023). CEP degeneration can therefore modify the local transport environment (Ren et al., 2023). Separately, experimental studies have linked CEP-cell injury to nuclear factor erythroid 2-related factor 2 (NRF2)-dependent regulation of ferritinophagy, the selective autophagic degradation of the iron-storage protein ferritin, and ferroptosis (Ma et al., 2024).

Disc degeneration involves interacting changes in tissue mechanics, structural integrity, and molecular transport. Human NP tissue exhibits altered mechanical behavior with degeneration and ageing (Iatridis et al., 1997). Experimental AF injury is associated with angiogenesis, inflammation, and fibrotic remodeling (Huang et al., 2025), whereas CEP degeneration can reduce solute diffusivity (Ren et al., 2023). Together with inflammatory matrix catabolism (Bermudez-Lekerika et al., 2022), these findings suggest a feedback process in which structural disruption and cellular dysfunction may reinforce one another.

These anatomical and transport characteristics are fundamental to the gut–disc axis. A circulating microbial metabolite or bacterial product cannot be assumed to influence the NP merely because it is detectable in feces or peripheral blood. Disc anatomy and CEP transport properties provide a basis for evaluating how circulating molecules might reach resident disc cells (Sun et al., 2020; Ren et al., 2023). Molecular properties, systemic pharmacokinetics, and tissue degeneration should therefore be examined as potential determinants of intradiscal exposure. AF injury-associated vascular remodeling suggests an additional possible route of access (Huang et al., 2025), but the transport of individual microbial metabolites or bacterial products through these pathways remains to be demonstrated.

### 2.2 Relative immune sequestration and its breakdown during degeneration

The healthy IVD, particularly the NP, is more accurately described as relatively immune-sequestered than immunologically inert. The absence of direct vascular and lymphatic access limits conventional immune surveillance, while the AF, CEPs, longitudinal ligaments, and dense extracellular matrix form a composite anatomical boundary separating the NP from the systemic immune compartment (Sun et al., 2020). The NP is derived from the embryonic notochord, and its anatomical isolation contributes to the concept of relative immune privilege (Sun et al., 2020).

The extracellular matrix itself contributes to immune exclusion. Human intervertebral disc aggrecan inhibits endothelial-cell adhesion and migration *in vitro*, indicating that the proteoglycan-rich matrix can directly oppose neovascularization (Johnson et al., 2005). The inhibitory effect of disc aggrecan on endothelial-cell adhesion and migration supports a direct contribution of matrix composition to vascular exclusion (Johnson et al., 2005). Anatomical isolation and restricted molecular transport provide additional constraints on tissue access (Ren et al., 2023).

Molecular mechanisms complement this anatomical sequestration. Fas ligand (FasL) expression has been demonstrated in disc cells (Takada et al., 2002). Functional studies using human NP cells further showed FasL-associated apoptosis of macrophages and CD8-positive T cells (Liu et al., 2013), as well as Fas-mediated apoptosis of vascular endothelial cells (Sun et al., 2013). These findings support potential roles for FasL in immune exclusion and the maintenance of an avascular disc environment. FasL expression decreases as human NP degeneration progresses, suggesting that failure of this checkpoint weakens the disc’s ability to exclude infiltrating lymphocytes and vascular cells (Kaneyama et al., 2008). Nevertheless, immune sequestration should not be interpreted as an absence of intrinsic inflammatory competence. NP, AF, and CEP cells can produce cytokines, chemokines, damage-associated molecular patterns, angiogenic factors, and matrix-degrading enzymes without leukocyte infiltration (Bermudez-Lekerika et al., 2022). Catabolic disc-cell activity may therefore precede, and subsequently promote, immune-cell entry.

Structural disruption marks a transition from relative immune sequestration to pathological immune exposure. Structural disruption can compromise the anatomical separation of the NP from surrounding tissues (Sun et al., 2020). Experimental AF injury additionally promotes angiogenic and inflammatory remodeling (Huang et al., 2025). AF injury also promotes vascular endothelial growth factor signaling, angiogenesis, inflammatory-cell entry, and fibrotic remodeling, reinforcing the concept that structural repair and restoration of immune separation are biologically linked (Huang et al., 2025). Vascular remodeling and hematogenous macrophage recruitment support increased communication with the systemic immune compartment (Yamamoto et al., 2022; Huang et al., 2025). Whether these changes also facilitate entry of particular microbial products or metabolites remains a testable hypothesis.

Human histological evidence supports this barrier-breach model. Macrophages accumulate within degenerated discs, particularly around structurally defective regions and damaged endplates. Nakazawa et al. identified increasing accumulation of macrophage phenotypes with advancing human disc degeneration (Nakazawa et al., 2018). Yamamoto et al. further demonstrated the distribution and polarization of hematogenous macrophages during disease progression, supporting recruitment from the systemic circulation rather than the presence of a uniform resident macrophage population in the healthy NP (Yamamoto et al., 2022). Human macrophage activation encompasses a spectrum of transcriptional states rather than a strict M1/M2 dichotomy (Xue et al., 2014). Accordingly, M1/M2 terminology is used here as a simplified description and not as a complete classification of intradiscal myeloid heterogeneity.

Exposure of NP components may additionally provoke antigen-specific immunity. Experimental immunization against NP antigens accelerated puncture-induced degeneration in rabbits (Bonilla et al., 2024). These observations support the capacity of previously sequestered NP components to participate in pathogenic immune responses after structural injury. They do not, however, establish autoimmunity as the initiating event in all forms of IDD. In many cases, mechanical damage, matrix catabolism, cellular senescence, or endplate dysfunction probably precedes overt immune exposure.

Barrier failure also modifies the interpretation of microbial findings in the disc. Detection of bacterial DNA in disc tissue does not by itself establish viable colonization or intestinal origin, particularly when contamination and analytical instability remain unresolved (Mengis et al., 2024; Fierer et al., 2025). Evidence for bacterial EV-mediated effects should be evaluated separately from sequencing evidence for a resident disc microbiome (Guan et al., 2026). The most defensible current model is therefore that degeneration creates an increasingly permissive interface through which systemic immune and microbial signals may influence disc cells, while the extent, route, and biological consequences of such exposure remain dependent on anatomical location and disease stage.

Anatomical isolation, matrix-associated inhibition of endothelial-cell migration, and FasL-related mechanisms contribute to relative immune sequestration of the healthy disc (Liu et al., 2013; Sun et al., 2013). Degeneration-associated changes in CEP transport, FasL expression, and vascular remodeling indicate that these protective features can be altered during disease (Kaneyama et al., 2008; Ren et al., 2023; Huang et al., 2025). These changes provide the anatomical basis for the gut–disc axis: intestinal dysbiosis and microbial metabolites can affect the disc only if their systemic signals reach a susceptible tissue compartment. Following barrier disruption, resident disc cells and recruited immune cells may become more responsive to systemic microbial and metabolic signals. Potential downstream mechanisms include pattern-recognition signaling and inflammasomes, intracellular protein complexes that activate inflammatory caspases, together with mitochondrial, lipid, and iron-regulatory pathways. Their integration into a complete gut-to-disc causal pathway remains to be established. The anatomical barriers and potential transport routes underlying gut–disc communication are summarized in **Figure 1**.

**Figure 1:**
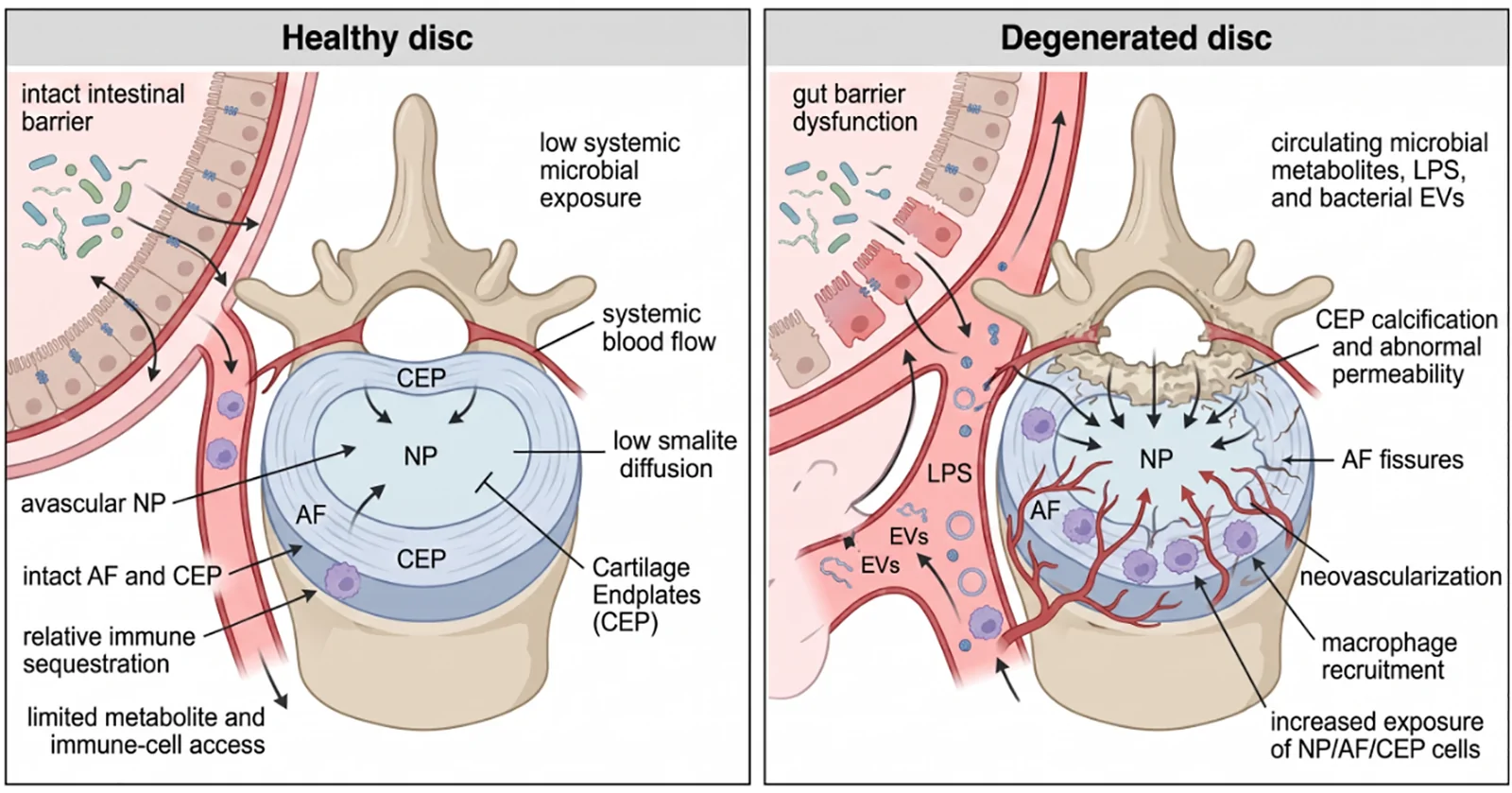
Anatomical barriers and potential routes of gut–disc communication. The healthy intervertebral disc is protected by a composite anatomical and molecular barrier comprising an intact annulus fibrosus, cartilage endplates, dense extracellular matrix, limited vascular and lymphatic access, and relative immune sequestration of the nucleus pulposus. Circulating microbial metabolites, lipopolysaccharide, bacterial extracellular vesicles, cytokines, and immune cells must traverse vertebral capillary–cartilage endplate pathways or vascularized regions of the outer annulus fibrosus to reach resident disc cells. During degeneration, cartilage-endplate calcification and dysfunction, annular fissuring, matrix disruption, and pathological neovascularization alter solute transport and may create permissive routes for systemic microbial and immune signals. These routes represent biologically plausible mechanisms of gut–disc communication and should not be interpreted as evidence of viable bacterial translocation or persistent intradiscal colonization. AF, annulus fibrosus; CEP, cartilage endplate; ECM, extracellular matrix; EVs, extracellular vesicles; IVD, intervertebral disc; LPS, lipopolysaccharide; NP, nucleus pulposus. Created in BioRender. DONG, Y. (2026). https://BioRender.com/uqsqxgx.

## 3 Immune–metabolic relay of the gut–disc axis

The gut–disc axis is considered here as a proposed framework in which intestinal dysbiosis, defined as an alteration in microbial community composition or function, may influence disc homeostasis through microbial products, circulating metabolites, and host immune responses. Individual components of this framework are supported by different study designs, but their integration into a sequential causal pathway remains incompletely demonstrated. Three non-mutually exclusive routes have been proposed: translocation of bacteria or bacterial components following disruption of the intestinal barrier, modulation of mucosal and systemic immunity, and altered production or absorption of microbiota-derived metabolites that influence distant tissues (Li et al., 2022). Human microbiome and metabolomic studies provide associative evidence (Rajasekaran et al., 2024; Liu J. et al., 2025), Mendelian randomization offers genetic inference (Ge et al., 2025), and paired fecal–disc multi-omics identifies tissue-associated microbial signals (Liu et al., 2026a). These approaches support different components of the proposed framework but do not establish an equivalent level of causality.

### 3.1 From gut dysbiosis to microbial signal exposure in the disc

Initial clinical studies have identified distinct gut microbial configurations in patients with degenerative disc disease. In a case–control study involving 40 patients with disc degeneration and 20 healthy volunteers, the degenerative group exhibited altered microbial community structure, depletion of beneficial short-chain fatty acid (SCFA)-producing bacteria, and increased circulating C-reactive protein and fatty acid-binding protein 5. These alterations were particularly evident in patients with Modic changes, magnetic resonance imaging signal abnormalities in the vertebral endplates and adjacent bone marrow. In this subgroup, Bifidobacterium and Ruminococcus were depleted, whereas several putative pathobionts were enriched (Rajasekaran et al., 2024). A subsequent study integrating 16S rDNA sequencing and untargeted fecal metabolomics similarly detected increased abundances of Proteobacteria and Fusobacteriota, together with an enrichment of lipid and lipid-like metabolites associated with oxidative stress and tissue degradation, in patients with intervertebral disc degeneration (IDD) (Liu J. et al., 2025). Gut dysbiosis has also been associated with lumbar degenerative spondylolisthesis, although this phenotype incorporates degeneration of both the disc and facet joints and should not be considered equivalent to isolated IDD (Aboushaala et al., 2024).

Genetic studies provide additional, albeit indirect, evidence. Mendelian randomization identified several microbial taxa associated with intervertebral disc disorders, including potentially protective members of the Erysipelotrichaceae family (Ge et al., 2025). However, a more recent analysis found no statistically supported mediation by the panel of circulating inflammatory cytokines examined, indicating that the gut–disc connection may not be explained solely by generalized cytokinemia (Yan et al., 2026). This negative mediation result is important because it redirects attention toward alternative routes, including microbial metabolites, local immune activation, bacterial extracellular vesicles, and direct microbe–host interactions.

The strongest currently available evidence for shared gut–disc microbial signatures has emerged from paired fecal and disc multi-omics analysis. However, shared signatures do not by themselves establish viable bacterial translocation or sustained colonization. In a paired fecal–disc study of 113 surgical patients, seven bacterial species were detected in both sample types, and the relative abundances of Phocaeicola vulgatus and Bacteroides thetaiotaomicron were associated with degeneration severity (Liu et al., 2026a). In the human single-cell transcriptomic analysis, disc chondrocyte-like cells with a high P. vulgatus signal showed increased transcript levels of *TLR4*, *MYD88*, *NFKB1*, and *RELA*, together with increased expression of genes associated with matrix degradation and fibrosis. Cell–cell communication analysis associated microbial signals with inferred chemokine, angiogenic, and profibrotic communication among disc cells and stromal or immune populations (Liu et al., 2026a). These computational associations do not establish that microbial exposure directly caused the inferred signaling changes. These findings provide a biologically coherent link between a gut-associated bacterium, lipopolysaccharide (LPS)-responsive signaling, metabolic reprogramming, and remodeling of the disc immune microenvironment.

Nevertheless, the precise route by which intestinal microbes or their products reach the disc remains incompletely resolved. Gut barrier dysfunction and metabolic endotoxemia are plausible mechanisms, but direct measurements of intestinal permeability and circulating endotoxin remain limited in IDD cohorts. Moreover, microbial detection within low-biomass disc tissue is vulnerable to contamination and analytical variation; sequencing signals alone do not establish viable colonization or intestinal origin (Mengis et al., 2024; Fierer et al., 2025). Thus, current evidence supports microbial signal exposure more strongly than it proves sustained bacterial invasion of the IVD. **Table 1** summarizes human association studies, experimental investigations, and early clinical interventions relevant to the gut–disc axis, together with their study designs and principal limitations.

**Table 1:** Current clinical and experimental evidence supporting the gut–disc axis in intervertebral disc degeneration.

Study	Design/model	Microbial exposure or intervention	Principal disc-related findings	Evidence level	Major limitation
Rajasekaran et al. (2024)	Human case–control study (40 patients with disc degeneration; 20 controls)	Fecal microbiome profiling	Altered microbial community structure, depletion of SCFA-producing taxa, and associations with CRP, FABP5, and Modic changes	Human association	Small, cross-sectional cohort; residual dietary, medication, and metabolic confounding
Liu et al. (Liu J. et al., 2025)	Human 16S rDNA sequencing plus untargeted fecal metabolomics	Gut microbial and fecal metabolite signatures	Increased Proteobacteria and Fusobacteriota with enrichment of lipid-related metabolites in IDD	Human multi-omics association	Fecal measurements only; no direct assessment of intradiscal exposure or target engagement
Ge et al. (2025)	Two-sample Mendelian randomization	Genetically instrumented microbial taxa	Several taxa showed potential protective or detrimental associations with intervertebral disc disorders	Genetic causal inference	Limited taxonomic resolution; dependent on instrument validity, ancestry, and horizontal pleiotropy
Guan et al. (2026)	Clinical association plus microbiota-depleted mice and multiple IDD models	A. muciniphila, bacterial EVs, and B2UKX5	A. muciniphila-derived EVs and B2UKX5 attenuated degeneration and improved ECM-related phenotypes	Direct preclinical causality with human correlation	Human therapeutic efficacy, long-term safety, biodistribution, and manufacturing reproducibility remain untested
Aboushaala et al. (2024)	Human symptomatic lumbar degenerative spondylolisthesis cohort	Fecal gut microbiome	Gut dysbiosis was associated with symptomatic degenerative spondylolisthesis	Human association	Phenotype includes disc and facet-joint degeneration and is not equivalent to isolated IDD
Yan et al. (2026)	Mendelian randomization with cytokine mediation analysis	Genetically instrumented microbial taxa and circulating cytokines	Microbial associations with IDD were not statistically mediated by the evaluated circulating cytokine panel	Genetic mediation analysis	Indirect evidence; limited mediator panel and no identification of the responsible microbial product or target cell
Liu et al. (2026a)	Paired fecal–disc multi-omics in 113 surgical patients	Shared fecal and disc microbial signatures, including P. vulgatus	Higher P. vulgatus signals were associated with increased expression of TLR4–MYD88–NF-κB-related transcripts and inferred immune, angiogenic, and fibrotic communication programs; direct pathway activation by the organism was not established.	Human tissue-level multi-omics	Operative, advanced-disease cohort; low-biomass contamination risk; shared signatures do not prove viable translocation
Wang et al. (2024)	Rat puncture-induced IDD model	Sanbi decoction with microbiome and serum-metabolome modulation	Altered microbial taxa and serum metabolites coincided with lower cytokines, improved ECM homeostasis, and preserved disc structure	Preclinical intervention	Multicomponent formulation; no single causal strain or metabolite was identified
Wang et al. (2023)	Rat IDD model and LPS-stimulated NP cells	Qiangjin Zhuanggu Qufeng mixture	Modified intestinal microbial and lipid-metabolic profiles while suppressing NLRP3-associated NP-cell pyroptosis	Preclinical intervention	Multicomponent formulation and parallel effects prevent attribution to a defined microbial metabolite
Ni et al. (2024)	Rat IDD model with host-gene manipulation and FMT	Microbiota generated after *Mettl3* silencing	FMT transferred part of the modified senescence and inflammatory phenotype to recipient animals	Transfer-based preclinical evidence	Donor microbiota resulted from host genetic manipulation and does not establish nonspecific FMT efficacy
Cheng et al. (2024)	Rabbit IDD model with fecal microbiome and metabolome profiling	Disease-associated microbial and metabolic changes	IDD was accompanied by altered microbial diversity and fecal pathways involving amino-acid, vitamin, and nucleotide metabolism	Animal multi-omics association	No causal strain, circulating mediator, disc exposure, or pathway-specific rescue
Lan et al. (2025)	Human metagenomic case–control study of Modic changes	Fecal microbial taxa and functional pathways	Reduced microbial diversity and discriminative taxa/pathways correlated with CRP and Pfirrmann grade	Human metagenomic association	Cross-sectional Modic-change phenotype; modest cohort and no external treatment-response validation
Mengis et al. (2024)	Human low-biomass disc microbiome replication study	Disc-tissue microbial sequencing with contamination controls	Failure to robustly reproduce previous disc microbiome signatures highlighted major analytical instability	Methodological evidence	Does not exclude transient microbial products or EVs; emphasizes contamination rather than establishing a gut–disc mechanism
Jensen et al. (2019)	Randomized, double-blind, placebo-controlled trial; 89 patients with chronic low back pain and Modic changes	Oral Lactobacillus rhamnosus GG	No significant benefit for predefined disability or combined back-and-leg pain outcomes	Completed clinical intervention	Does not establish structural modification of IDD
Nian et al. (2025)	Multicenter randomized placebo-controlled trial protocol	Oral Bifidobacterium adolescentis	Clinical, imaging, and microbiome-related outcomes specified; efficacy results not reported in the cited publication	Clinical trial protocol	Protocol cannot establish treatment efficacy

Evidence levels describe the directness of the gut–disc relationship rather than overall study quality. CRP, C-reactive protein; ECM, extracellular matrix; EV, extracellular vesicle; FABP5, fatty acid-binding protein 5; FMT, fecal microbiota transplantation; IDD, intervertebral disc degeneration; NF-κB, nuclear factor-κB; NP, nucleus pulposus; SCFA, short-chain fatty acid; TLR4, Toll-like receptor 4.

### 3.2 Microbial metabolites as immunometabolic signals

Microbial metabolites may connect intestinal ecology with disc homeostasis without requiring viable bacteria to enter the IVD. SCFAs produced through microbial fermentation can influence immune-cell function through receptor-dependent and epigenetic mechanisms (Liu XF. et al., 2023). For example, butyrate promoted colonic regulatory T-cell differentiation and increased histone acetylation at the *Foxp3* locus (Furusawa et al., 2013). A separate atherosclerosis study implicated GPR109A signaling in butyrate-mediated suppression of macrophage pyroptosis (Hua et al., 2025). These findings establish mechanistic examples outside the disc rather than direct evidence of an intradiscal SCFA pathway. The depletion of SCFA-producing taxa observed in patients with disc degeneration therefore provides a plausible mechanism by which dysbiosis could weaken systemic inflammatory restraint (Rajasekaran et al., 2024). However, direct evidence that physiological concentrations of gut-derived SCFAs reach the IVD and regulate disc-cell pyroptosis or ferroptosis is not yet available.

Human disc metabolomic profiling nevertheless demonstrates that the degenerating IVD is metabolically responsive. Compared with non-degenerated cells, degenerated NP and AF cells exhibited altered lipid profiles, including reduced levels of several lipid classes and increased bile acids and ceramides (Francisco et al., 2023). Gene-expression profiling of human NP and AF cells also identified alterations in inflammatory and matrix-remodeling transcripts, including *IL1B*, *IL6*, *CXCL8*, *TNF*, *NOS2*, *PTGS2*, *MMP9*, and *MMP13 (*Francisco et al., 2023). These findings support an association between altered cellular lipid profiles and inflammatory or catabolic gene expression, but do not by themselves demonstrate regulated cell death. The origin of the accumulated bile acids was not established, and they should therefore not automatically be classified as microbiota-derived in this setting. These findings demonstrate an association between altered cellular lipid profiles and inflammatory features, but do not establish metabolite origin or the direction of causality (Francisco et al., 2023).

Intervention studies provide further support for microbiota–metabolite coupling. In a rat puncture model, Sanbi decoction altered the abundances of several intestinal bacterial taxa and shifted serum concentrations of α-muricholic acid, 5-hydroxyindoleacetic acid, kynurenine, and other lipid- and amino-acid-related metabolites. These changes coincided with lower circulating TNF-α, IL-1β, and IL-6, improved extracellular matrix (ECM) homeostasis, and preservation of disc structure (Wang et al., 2024). Similarly, Qiangjin Zhuanggu Qufeng mixture altered Enterobacteriaceae- and Clostridium-related microbial profiles and lipid metabolism while suppressing pyroptosis associated with NLR family pyrin domain-containing 3 (NLRP3), an intracellular inflammasome sensor, in NP cells (Wang et al., 2023). Because both interventions contain multiple bioactive compounds, these studies demonstrate coordinated microbiome–metabolome–disc modulation but do not identify a single causal microbial metabolite.

Computational analyses have nominated butyrate, propionate, acetate, succinate, trimethylamine N-oxide, and indole-3-propionic acid as candidate metabolites associated with IDD-related networks. Predicted protein targets included IL-6, TLR4, CXCL8, and JUN, alongside pathways related to oxidative stress and lipid metabolism (Liu et al., 2026b). These predictions are hypothesis-generating rather than experimental validation. Among microbiota-associated metabolites, urolithin A has been shown to preserve mitochondrial function and reduce oxidative stress-induced senescence in NP-derived mesenchymal stem cells through the SIRT1/PGC-1α pathway (Shi et al., 2021). Although this finding supports the responsiveness of disc-resident cells to a microbiota-derived metabolite, its direct effects on NP-cell pyroptosis and ferroptosis remain to be established.

### 3.3 Macrophages as central immune–metabolic intermediaries

Macrophages provide a potential link between systemic inflammatory signals and disc-cell injury. LT-α-induced glycolytic reprogramming promoted inflammatory macrophage polarization and generated a conditioned environment that impaired NP-cell function (Qiu et al., 2025). Similarly, conditioned medium from LPS/IFN-γ-polarized macrophages aggravated oxidative stress and matrix-related injury in NP cells (Hou X. et al., 2022). These experimental phenotypes are useful for mechanistic analysis, although macrophage activation encompasses a broader spectrum than the binary M1/M2 classification (Xue et al., 2014).

Human single-cell transcriptomic analyses identified a myeloid-derived suppressor cell population with high *LCN2* expression. These cells belong to a heterogeneous group of myeloid cells with immunosuppressive activity. Subsequent functional experiments supported a potentially protective role in IDD, involving ANXA1-associated anti-inflammatory activity and modulation of macrophage polarization (Zhang C. et al., 2025). By contrast, M1 macrophage-derived extracellular vesicles delivered LCN2 to NP cells, activated NF-κB, enhanced p21/p53-associated senescence, increased MMP13, and reduced type II collagen (Fan et al., 2024). The apparently opposing effects of LCN2 emphasize that the biological activity of an immune mediator depends on its cellular source, molecular context, cargo composition, and disease stage rather than on expression level alone.

Metabolic reprogramming further stabilizes inflammatory macrophage states. LT-α increased glucose uptake, lactate production, and aerobic glycolysis in macrophages through NF-κB, thereby enhancing M1 polarization and generating a conditioned environment that impaired NP-cell proliferation and migration and increased apoptosis (Qiu et al., 2025). Consistently, medium derived from LPS/IFN-γ-polarized macrophages aggravated oxidative stress, inflammatory injury, and matrix loss in NP cells, whereas inhibition of M1 polarization attenuated these effects (Hou X. et al., 2022). The association between intradiscal P. vulgatus signals and inferred disc-cell–macrophage communication provides an additional observational link to this circuit, although direct microbial causation remains unresolved (Liu et al., 2026a).

Other immune-cell populations may extend this relay. SCFAs influence multiple immune-cell populations (Liu XF. et al., 2023), and butyrate has been experimentally shown to promote colonic regulatory T-cell differentiation (Furusawa et al., 2013). Whether reduced SCFA availability alters Th17/Treg balance within degenerative discs remains hypothetical. Reduced SCFA availability could theoretically weaken Treg-mediated restraint and favor IL-17-dominant responses. However, the currently available IDD-specific evidence does not establish a direct sequence linking a defined gut metabolite to Th17/Treg imbalance within the human disc. Likewise, the roles of neutrophils, B cells, bacterial extracellular vesicles, and complement in the gut–disc axis remain insufficiently characterized. These pathways should therefore be presented as plausible components of multicellular immune amplification rather than established causal mechanisms.

An additional layer of bidirectionality is suggested by experiments linking host molecular changes to gut microbial composition. In a rat IDD model, *Mettl3* silencing reduced *Tlr2* mRNA stability and NP-cell senescence, suppressed factors associated with the senescence-associated secretory phenotype (SASP), and altered gut microbial composition. SASP refers to the secretory program of senescent cells, which includes inflammatory mediators and tissue-remodeling factors. Fecal microbiota transplantation transferred part of the altered phenotype to recipient animals (Ni et al., 2024). This finding suggests that the gut–disc axis may operate as a reciprocal system in which disc-associated inflammatory programs and host epigenetic regulation reshape the intestinal ecosystem, rather than as a strictly unidirectional gut-to-disc pathway.

### 3.4 Coupling microbial signaling to pyroptosis and ferroptosis

Pyroptosis represents a mechanistically direct interface between microbial recognition and inflammatory disc-cell death. In canonical inflammasome signaling, activated caspase-1 cleaves GSDMD, enabling gasdermin-mediated membrane permeabilization and pyroptotic death (Shi et al., 2015). Intracellular LPS can be directly sensed by human caspase-4 and murine caspase-11, triggering noncanonical inflammasome activation and pyroptotic cell death (Shi et al., 2014). Within disc cells, CD14-dependent LPS recognition activates NF-κB, caspase-1, and IL-1β processing. Interference with the LPS-binding region of CD14 suppressed these responses, supporting the functional relevance of microbial pattern recognition in the IVD (Alad et al., 2024).

The paired human fecal–disc study identified an association between P. vulgatus signals and increased expression of transcripts related to TLR4–MYD88 signaling (Liu et al., 2026a).This provides observational context but does not demonstrate that gut-derived P. vulgatus or its LPS directly activates this pathway in disc cells. Downstream experimental evidence shows that microbiota-associated interventions can suppress NP-cell pyroptosis: Qiangjin Zhuanggu Qufeng mixture reduced NLRP3 expression and pyroptosis-associated mediators in LPS-stimulated NP cells while concurrently altering intestinal microbial and serum metabolic profiles *in vivo (*Wang et al., 2023). Mitochondrial quality control provides an additional checkpoint. IL-1β-induced mitochondrial reactive oxygen species (mtROS) promoted NLRP3 activation and NP-cell pyroptosis. In contrast, SIRT1-dependent PINK1/parkin-mediated mitophagy, the selective autophagic removal of mitochondria, restored mitochondrial homeostasis and suppressed NLRP3 inflammasome activation (Ma et al., 2022). Microbial metabolites capable of regulating SIRT1, mitophagy, or cellular redox balance could therefore influence the threshold for pyroptosis, although this connection remains incompletely tested in IDD.

Ferroptosis may represent the complementary metabolic arm of the relay. Ferroptosis is driven by iron-dependent lipid peroxidation and is restrained by antioxidant defenses that include GPX4 (Dixon et al., 2012; Yang et al., 2014). Bioinformatic and experimental analyses have identified concurrent alterations in ferroptosis-related genes and immune-cell infiltration in degenerated NP tissue, including increased NCOA4 and PCBP1 and reduced GPX4-related protection (Liu XW. et al., 2023). In cartilage endplate chondrocytes, insufficient NRF2 activation enhanced NCOA4-mediated ferritinophagy, increased ferrous iron accumulation, mitochondrial injury, ECM degradation, and ferroptotic cell death (Ma et al., 2024). TNF-α further aggravated this iron-handling defect, illustrating how inflammatory cytokines can lower the threshold for ferroptosis.

Disc-specific studies link macrophage-associated inflammatory conditions and altered iron handling to ferroptotic injury (Ma et al., 2024), while human disc-cell profiling identifies changes in lipid and bile-acid-related metabolites (Dou et al., 2023; Francisco et al., 2023). Separately, SCFAs, tryptophan derivatives, and urolithins modulate ferroptosis-related processes in non-disc models (Dong et al., 2025; Li Y. et al., 2025; Chen et al., 2026; Wang X. et al., 2026). These findings motivate a possible gut–disc connection but do not establish that microbial-metabolite depletion directly causes ferroptosis within the IVD. Nevertheless, among the studies examined, direct demonstration that a defined gut microbial metabolite controls ferroptosis in disc cells remains lacking. A testable hypothesis is that dysbiosis may modify systemic immune and metabolic conditions in ways that increase disc-cell susceptibility to ferroptosis. The reviewed studies do not establish this complete causal sequence or identify a dominant gut-derived metabolite responsible for intradiscal ferroptotic injury.

Together, clinical microbial associations (Rajasekaran et al., 2024; Liu J. et al., 2025; Liu et al., 2026a) and experimental studies of LPS recognition, mitochondrial quality control, and ferroptotic injury in disc cells (Ma et al., 2022; Alad et al., 2024; Ma et al., 2024) support the plausibility of an immune–metabolic relay. The complete sequence from intestinal dysbiosis to intradiscal pyroptosis or ferroptosis remains a proposed model rather than a fully validated causal pathway. Defining the directionality, tissue concentrations, cellular receptors, and stage-specific effects of individual microbial metabolites will be essential before this framework can be translated into biomarker-guided or metabolite-based therapy. The proposed immune–metabolic relay linking gut dysbiosis to pyroptosis and ferroptosis is illustrated in **Figure 2**.

**Figure 2:**
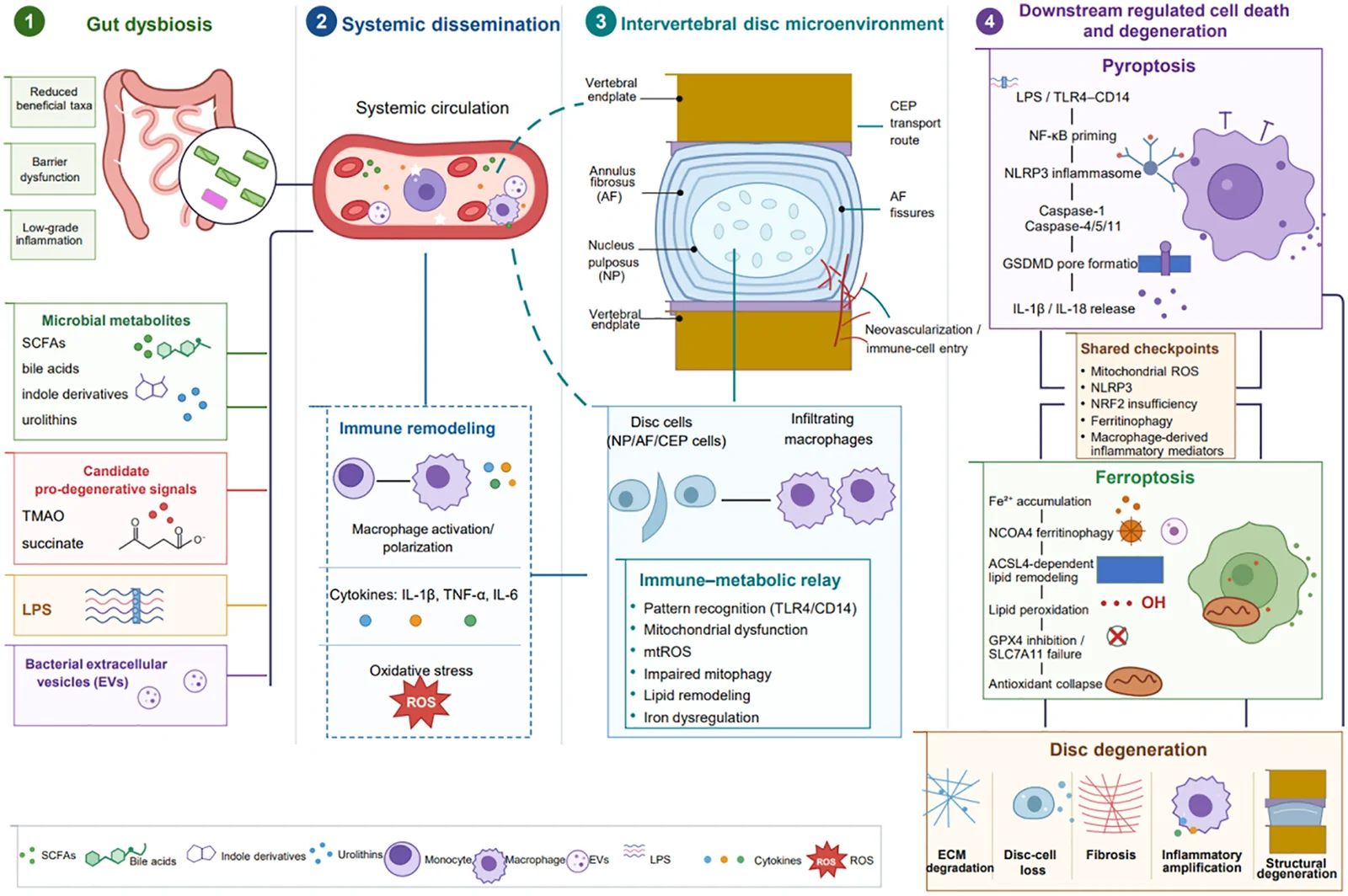
Immune–metabolic relay linking gut dysbiosis to pyroptosis and ferroptosis in intervertebral disc degeneration. Gut dysbiosis is characterized by depletion of beneficial taxa, intestinal-barrier dysfunction, and low-grade inflammation. Microbial metabolites, candidate pro-degenerative signals, lipopolysaccharide, and bacterial extracellular vesicles may enter the systemic circulation and promote immune remodeling, macrophage activation, cytokine production, and oxidative stress. Circulating signals may reach susceptible intervertebral disc compartments through cartilage-endplate transport routes, annular fissures, or neovascularized regions. Disc cells and infiltrating macrophages integrate these signals through TLR4/CD14-mediated pattern recognition, mitochondrial dysfunction, mitochondrial reactive oxygen species production, impaired mitophagy, lipid remodeling, and iron dysregulation. Activation of the NLRP3–caspase–GSDMD pathway promotes pyroptosis and IL-1β/IL-18 release, whereas Fe²^+^ accumulation, NCOA4-mediated ferritinophagy, ACSL4-dependent lipid remodeling, lipid peroxidation, and failure of the SLC7A11–GSH–GPX4 axis promote ferroptosis. Shared mitochondrial, redox, inflammatory, and iron-regulatory checkpoints amplify extracellular-matrix degradation, disc-cell loss, fibrosis, inflammation, and structural degeneration. Solid arrows denote mechanisms experimentally demonstrated in the tissue or model specified in the image. Dashed arrows denote proposed connections, including unverified transport of individual microbial metabolites into the disc and extrapolation of non-disc mechanisms to disc cells. Human associations are identified separately and do not imply causality. AF, annulus fibrosus; ACSL4, acyl-CoA synthetase long-chain family member 4; CEP, cartilage endplate; ECM, extracellular matrix; EVs, extracellular vesicles; GSDMD, gasdermin D; GPX4, glutathione peroxidase 4; GSH, glutathione; IDD, intervertebral disc degeneration; IL, interleukin; LPS, lipopolysaccharide; mtROS, mitochondrial reactive oxygen species; NF-κB, nuclear factor-κB; NLRP3, NOD-like receptor family pyrin domain-containing 3; NP, nucleus pulposus; NRF2, nuclear factor erythroid 2-related factor 2; ROS, reactive oxygen species; SCFAs, short-chain fatty acids; SLC7A11, solute carrier family 7 member 11; TLR4, Toll-like receptor 4; TMAO, trimethylamine N-oxide. Created in BioRender. DONG, Y. (2026). https://BioRender.com/vr3dm8m.

## 4 Microbial metabolites at the pyroptosis–ferroptosis interface in intervertebral disc degeneration

Microbial metabolites provide a mechanistically attractive link between intestinal dysbiosis and regulated cell death in the intervertebral disc. Microbiota-dependent metabolites contribute to the circulating metabolome (Wikoff et al., 2009), and specific metabolites can regulate receptor signaling and inflammatory programs in tissue macrophages (Hua et al., 2025; Luo et al., 2025). These observations provide biological plausibility for distant effects but do not establish exposure or activity within the disc. Network pharmacology and molecular docking have nominated butyrate, propionate, acetate, succinate, trimethylamine N-oxide (TMAO), and indole-3-propionic acid (IPA) as candidate mediators of the gut–disc axis, with IL-6, TLR4, CXCL8, and JUN emerging as highly connected host targets involved in inflammation, oxidative stress, lipid metabolism, and extracellular matrix remodeling (Liu et al., 2026b). However, these computational associations do not establish tissue exposure, target engagement, or causality in the intervertebral disc.

This distinction is particularly important when considering pyroptosis and ferroptosis. Pyroptosis is an inflammatory, membrane-lytic form of regulated cell death involving inflammasome activation, inflammatory caspases, and gasdermin-mediated pore formation, whereas ferroptosis is driven by uncontrolled iron-dependent phospholipid peroxidation and failure of antioxidant defense systems (Mishima et al., 2025; Wu W. et al., 2026). Experimental studies implicate pyroptosis and ferroptosis in disc-cell injury (Yang et al., 2021; Ma et al., 2022; Ma et al., 2024). However, the studies reviewed here provide limited direct evidence connecting a defined gut-derived metabolite, verified intradiscal exposure, and either death program. To avoid conflating tissue relevance with causal strength, the evidence is classified into four categories: human observational or genetic evidence; experimental evidence obtained in disc cells or IDD models; mechanistic evidence from non-disc tissues; and computational predictions or untested hypotheses. Within disc-specific experiments, studies demonstrating a microbial mediator are distinguished from those investigating local host metabolism or downstream cell-death pathways. These categories describe the source and applicability of evidence and do not constitute a formal study-quality grading system. The evidence supporting individual metabolites and their relevance to the pyroptosis–ferroptosis interface is summarized in **Table 2**.

**Table 2:** Microbial metabolites and local metabolic analogues implicated in the pyroptosis–ferroptosis interface.

Metabolite/class	Proposed source	Experimental tissue/model	Key target or pathway	Reported effect	Directness for IDD	Major caveat
SCFAs (general) (Liu XF. et al., 2023)	Microbial fermentation of dietary substrates	Immune-system studies across multiple tissues	FFAR signaling, HDAC inhibition, immune-cell metabolic remodeling	Context-dependent regulation of TLR signaling, NLRP3, macrophages, neutrophils, and adaptive immunity	Mechanistic background only	Physiological circulating and intradiscal concentrations have not been linked to disc-cell death
Butyrate (Hua et al., 2025)	Gut microbial fermentation	Macrophages in atherosclerosis models	GPR109A–GSDMD	Reduced LPS leakage and suppressed macrophage pyroptosis	Extra-disc mechanistic evidence	Receptor expression and equivalent activity in disc cells or infiltrating disc macrophages remain unknown
Propionate (Yao et al., 2025)	Gut microbial fermentation	Experimental colitis	HIF-1α-associated TFR1/FTH1 regulation	Restored iron homeostasis, promoted reparative macrophage polarization, and inhibited ferroptosis	Extra-disc mechanistic evidence	Colon-specific exposure and inflammatory context may not translate to the disc
Sodium butyrate (Chen et al., 2026)	Microbial metabolite/pharmacologic supplementation	Inflammatory bowel disease models	SIRT1–SLC7A11–GPX4 and ACSL4	Attenuated tissue injury and was associated with reduced macrophage ferroptosis	Extra-disc mechanistic evidence	Cell-specific contribution and effective intradiscal dose are unresolved
Sodium butyrate (Liu L. et al., 2025)	Microbial metabolite/pharmacologic supplementation	Colorectal cancer cells	NCOA4–FTH1 ferritinophagy	Increased Fe2+, lipid ROS, and ferroptotic death	Context-dependent counterexample	Cancer-cell metabolism and high-dose exposure limit extrapolation to nonmalignant disc cells
Lithocholic acid [66.69]	Microbiota-modified secondary bile acid	Experimental colitis; neutrophils and macrophages	NLRP3–GSDMD and caspase-1/IL-1β signaling	Reduced NET formation, M1 polarization, inflammasome activation, and inflammatory injury	Extra-disc mechanistic evidence	No direct measurement of LCA exposure or receptor engagement in the IVD
Taurodeoxycholic acid (Ruan et al., 2026)	Host–microbial bile-acid pool	LPS-driven liver injury and macrophages	Caspase-11–GSDMD noncanonical pyroptosis	Reduced GSDMD cleavage, cytokine release, membrane permeability, and macrophage pyroptosis	Extra-disc mechanistic evidence	Effects on human caspase-4/5 and disc cells remain untested
Conjugated primary bile acids (Fan et al., 2026)	Host-derived bile acids influenced by enterohepatic metabolism	Cholangiocytes and macrophage recruitment	Mitochondrial permeability transition, oxidized mtDNA–STING, inflammasome signaling	Promoted senescence-associated inflammatory signaling and nonlethal pyroptotic responses	Extra-disc mechanistic evidence	Bile-acid species have divergent actions and cannot be generalized as a single class
Indole-3-propionic acid (Luo et al., 2025; Wu R. et al., 2025; Liu et al., 2026b)	Microbial tryptophan metabolism	Computational IDD analysis; bone-loss and liver-fibrosis models	NF-κB/NLRP3 and macrophage AHR signaling	Predicted IDD target interactions and reduced myeloid inflammatory/profibrotic programs in other tissues	Computational IDD plus extra-disc evidence	No direct disc exposure, receptor engagement, or regulated-cell-death rescue in IDD
Indole-3-carboxaldehyde (Miao et al., 2025)	B. longum-associated tryptophan metabolite	Macrophages in intestinal ischemia/reperfusion injury	HDAC3 and mitochondrial fatty-acid oxidation	Inhibited macrophage NLRP3 inflammasome formation	Extra-disc mechanistic evidence	Disc-cell responsiveness and pharmacokinetics remain unknown
Indole-3-pyruvic acid (Dong et al., 2025)	Host IL4I1-mediated tryptophan metabolism in the cited model; a gut microbial contribution was not established.	Macrophages after myocardial infarction	AHR–NRF2–FTH1–GPX4	Reduced lipid peroxidation and macrophage ferroptosis	Extra-disc mechanistic evidence	Metabolite origin is not exclusively microbial and disc-specific effects are untested
Urolithin A (Shi et al., 2021; Wang X. et al., 2026)	Microbial transformation of dietary ellagitannins	NP-derived mesenchymal stem cells; articular chondrocytes	SIRT1/PGC-1α and AMPK–mTOR–HIF-1α	Reduced NP-cell senescence and protected chondrocytes from inflammatory ferroptosis	Direct evidence in NP-derived cells for senescence; non-disc evidence in articular chondrocytes for ferroptosis.	Intradiscal bioavailability and direct NP/AF/CEP ferroptosis rescue remain unproven
Urolithin B (Li Y. et al., 2025)	Microbial transformation of dietary polyphenols	Inflammatory articular-chondrocyte model	FGFR3–NCOA4–FTH1	Suppressed ferritinophagy, restored iron homeostasis, and inhibited ferroptosis	Musculoskeletal indirect evidence	Articular chondrocytes are not equivalent to NP, AF, or CEP cells
Imidazole propionate (Park et al., 2025)	Gut microbial histidine metabolism	Systemic and brain exposure in neurodegeneration models	mTORC1-dependent metabolic and inflammatory signaling	Promoted neuroinflammation and degenerative pathology	Extra-disc candidate pro-degenerative signal	Pyroptosis and ferroptosis were not established as the principal execution pathways
TMAO (Li D. et al., 2025)	Microbial trimethylamine production followed by hepatic oxidation	Bioprosthetic-valve calcification and macrophages	Macrophage pyroptosis and inflammatory calcification	Promoted inflammatory injury and macrophage pyroptosis	Extra-disc candidate signal	No direct IDD, endplate, or intradiscal exposure data
Succinate (Liu et al., 2026b)	Host and microbial metabolism	Network pharmacology and molecular docking for IDD	Predicted IL-6, TLR4, CXCL8, JUN, and lipid-related networks	Nominated as a candidate gut–disc mediator	Computational prediction only	No causal strain–succinate–disc-cell-death pathway has been demonstrated
Lactate (Liu W. et al., 2026; Xia et al., 2026; Zhang Y. et al., 2026)	Predominantly local host glycolysis within the hypoxic disc	Macrophages; NP cells; rat IDD models	NLRP3 lactylation, SOD1-K123 lactylation, and catalytic conversion to alanine	Enhanced inflammasome/oxidative injury; lactate removal reduced NLRP3 activity and improved disc repair	Disc-specific evidence for local lactate-associated injury; NLRP3 lactylation demonstrated in a non-disc macrophage model.	Intradiscal lactate is not evidence of microbial origin
Glutarate (Yu et al., 2026)	Locally accumulated metabolite; gut origin not established	NP cells and experimental IDD	SUGCT-dependent NRF2 glutarylation and KEAP1 dissociation	Reduced iron overload, lipid peroxidation, and TNF-α-induced ferroptotic injury	Direct IDD evidence for a local metabolite	Protective local compensation should not be classified as a confirmed microbiota-derived signal

Directness for IDD” describes whether the cited findings were obtained in disc-related experimental systems, non-disc tissues, or computational analyses; it does not represent a formal assessment of study quality. Disc-specific effects do not establish a gut microbial origin. Lactate and glutarate are included as local metabolic examples, and their relevant intradiscal pools have not been established as gut-derived. ACSL4, acyl-CoA synthetase long-chain family member 4; AF, annulus fibrosus; AHR, aryl hydrocarbon receptor; CEP, cartilage endplate; FFAR, free fatty acid receptor; FTH1, ferritin heavy chain 1; GPX4, glutathione peroxidase 4; GSDMD, gasdermin D; HDAC, histone deacetylase; IDD, intervertebral disc degeneration; IVD, intervertebral disc; LCA, lithocholic acid; LPS, lipopolysaccharide; mtDNA, mitochondrial DNA; NET, neutrophil extracellular trap; NP, nucleus pulposus; NRF2, nuclear factor erythroid 2-related factor 2; ROS, reactive oxygen species; SCFA, short-chain fatty acid; SLC7A11, solute carrier family 7 member 11; STING, stimulator of interferon genes; TDCA, taurodeoxycholic acid; TFR1, transferrin receptor 1; TMAO, trimethylamine N-oxide.

### 4.1 Short-chain fatty acids: context-dependent control of inflammatory and iron-dependent cell death

Short-chain fatty acids (SCFAs), particularly acetate, propionate, and butyrate, are among the most extensively studied microbial metabolites in systemic immune regulation. Reported SCFA actions include receptor-dependent regulation of macrophage inflammation and intestinal barrier function (Hua et al., 2025), as well as epigenetic effects associated with colonic regulatory T-cell differentiation (Furusawa et al., 2013).Within the proposed gut–disc axis, loss of SCFA-producing bacteria could theoretically remove an important brake on systemic inflammation, whereas restoration of SCFA signaling may reduce exposure of the disc to endotoxin and inflammatory mediators. Nevertheless, direct measurements linking circulating SCFA concentrations to intradiscal cell-death activity have not yet been reported in the retrieved literature.

Evidence from macrophage-centered disease models provides a plausible route linking SCFAs to pyroptosis. In atherosclerosis models, Ligilactobacillus murinus and Lactobacillus johnsonii formed microbial networks with butyrate-producing bacteria, preserved intestinal barrier function, and reduced lipopolysaccharide leakage. Butyrate directly activated GPR109A and suppressed gasdermin D expression in macrophages, thereby limiting pyroptosis (Hua et al., 2025). This gut microbiota–butyrate–GPR109A–GSDMD pathway suggests that SCFAs may act at two levels: reducing the systemic delivery of pyroptotic stimuli and directly restraining the execution machinery of inflammatory macrophage death. Whether disc-infiltrating macrophages or resident disc cells express a functionally equivalent GPR109A–GSDMD circuit remains unknown.

SCFAs also regulate ferroptosis, although their actions are strongly dependent on dose and cellular context. In experimental colitis, propionate reduced inflammatory cytokine production, promoted reparative macrophage polarization, and altered the expression of transferrin receptor 1 and ferritin heavy chain 1 through an HIF-1α-associated mechanism. These effects restored iron homeostasis, supported epithelial regeneration, and inhibited ferroptosis (Yao et al., 2025). Sodium butyrate similarly attenuated intestinal injury while increasing SIRT1, SLC7A11, and GPX4 and decreasing ACSL4, with immunofluorescence evidence suggesting reduced macrophage ferroptosis (Chen et al., 2026). These observations overlap with disc-specific evidence implicating impaired antioxidant protection, ACSL4-related pathways, and macrophage-associated injury in ferroptosis (Yang et al., 2021; Dou et al., 2023; Feng et al., 2025). Such overlap supports further investigation but does not demonstrate that SCFAs regulate these pathways in disc cells.

However, butyrate should not be regarded as intrinsically anti-ferroptotic. In colorectal cancer cells, sodium butyrate promoted NCOA4–FTH1-dependent ferritinophagy, increased lysosomal and cytosolic Fe²^+^, enhanced lipid reactive oxygen species, and induced ferroptosis (Liu L. et al., 2025). The opposite effects observed in inflammatory tissue and cancer models indicate that butyrate activity depends on concentration, metabolic state, cell lineage, and ferritinophagic competence. Accordingly, extrapolation to the disc requires direct testing in nucleus pulposus (NP), annulus fibrosus, cartilage endplate, and infiltrating immune-cell populations under physiologically relevant concentrations. The currently available IDD-specific evidence is computational: butyrate, propionate, and acetate were predicted to interact with inflammation- and lipid-associated targets, but receptor engagement and functional effects on disc-cell pyroptosis or ferroptosis have not been experimentally confirmed (Liu et al., 2026b).

### 4.2 Bile acid metabolites: divergent regulation of inflammasome activation and mitochondrial injury

The effects of individual bile acids differ across experimental settings. Lithocholic acid (LCA) and taurodeoxycholic acid have been associated with suppression of inflammatory or pyroptosis-related responses in colitis and liver-injury models (Xu et al., 2025; Ruan et al., 2026). In contrast, conjugated primary bile acids can promote mitochondrial injury and inflammatory signaling involving stimulator of interferon genes (STING), an adaptor protein in cytosolic DNA-sensing pathways, in cholangiocytes (Fan et al., 2026). Bile acids should therefore be evaluated by molecular species and tissue context rather than treated as a uniformly protective class. In experimental colitis, administration of Odoribacter splanchnicus increased the microbiota-dependent production of lithocholic acid (LCA), preserved barrier integrity, and reduced neutrophil extracellular trap formation. LCA reproduced the protective effect and inhibited the NLRP3–GSDMD pathway, defining a microbiota–LCA–neutrophil axis that limits inflammatory tissue injury (Xu et al., 2025). A separate study showed that microbiome remodeling increased LCA abundance while reducing M1 macrophage polarization, caspase-1 activity, and IL-1β production (Zhong et al., 2026). Together, these observations suggest that LCA can suppress both neutrophil- and macrophage-mediated inflammasome amplification.

Bile acids may also regulate noncanonical pyroptosis. Taurodeoxycholic acid reduced caspase-11 activation, GSDMD cleavage, IL-1β and IL-18 release, membrane permeability, and macrophage pyroptosis in an LPS-driven liver-injury model (Ruan et al., 2026). Noncanonical pyroptosis requires intracellular access of LPS, which can be sensed by inflammatory caspases (Shi et al., 2014). The taurodeoxycholic-acid findings provide evidence from a murine caspase-11-associated liver-injury model (Ruan et al., 2026); equivalent effects on human caspase-4/5 signaling or on disc-resident and disc-infiltrating cells have not been established.

Conversely, bile acid accumulation can be detrimental. Conjugated primary bile acids induced mitochondrial permeability transition, mitochondrial damage, and leakage of oxidized DNA in cholangiocytes. The resulting STING-dependent senescence-associated secretory phenotype recruited macrophages and promoted inflammasome activation and non-lethal pyroptotic signaling (Fan et al., 2026). This contrasting result underscores that the biological direction of bile acid signaling depends on molecular identity and tissue context. Separate IDD studies implicate mitochondrial DNA release and STING-related signaling in disc-cell injury (Xu et al., 2026; Zhou H. et al., 2026). These findings identify a possible point of mechanistic overlap, but do not demonstrate that bile acids activate this pathway within the disc.

No study in the retrieved dataset directly established that LCA, taurodeoxycholic acid, or another microbiota-modified bile acid regulates pyroptosis or ferroptosis in the IVD. Consequently, bile acids should currently be framed as candidate systemic regulators rather than confirmed disc-active metabolites. Future experiments should quantify individual bile acid species in paired fecal, plasma, endplate, and disc samples and determine whether their effects are mediated through canonical bile acid receptors or through receptor-independent mitochondrial and membrane stress.

### 4.3 Tryptophan derivatives and urolithins: linking aryl hydrocarbon receptor signaling to inflammasome and ferroptosis control

Tryptophan-derived metabolites differ in both origin and biological activity. Microbial indoles can influence intestinal barrier function through pregnane X receptor signaling (Venkatesh et al., 2014). Other studies implicate histone deacetylase 3 (HDAC3), an enzyme involved in protein deacetylation, or the aryl hydrocarbon receptor (AHR), a ligand-activated transcription factor, in the actions of individual metabolites (Luo et al., 2025; Miao et al., 2025). The IL4I1-derived indole-3-pyruvic acid investigated in myocardial infarction should be distinguished from metabolites with an experimentally established gut microbial origin (Dong et al., 2025). Among the candidate gut-derived metabolites computationally associated with IDD, IPA showed particularly favorable predicted interactions with IL-6, TLR4, CXCL8, and JUN (Liu et al., 2026b). However, this prediction has not yet been validated in disc cells.

Evidence from bone and immune-cell models suggests that IPA may restrain inflammasome activity. In high-fat-diet-induced bone loss, IPA supplementation reduced systemic inflammation and osteoclast activation by suppressing NF-κB phosphorylation and decreasing NLRP3, ASC, and caspase-1 expression (Wu R. et al., 2025). Because osteoclast precursors and infiltrating disc macrophages share a monocyte-lineage origin, this result supports a potential role for IPA in suppressing myeloid inflammasome activation, although it does not demonstrate an equivalent response within the IVD.

Indole-3-carboxaldehyde provides an additional link between microbial metabolism, mitochondrial activity, and inflammasome assembly. A metabolite produced in association with Bifidobacterium longum, indole-3-carboxaldehyde inhibited macrophage inflammasome formation by targeting HDAC3 and altering mitochondrial fatty acid oxidation (Miao et al., 2025). Gut-derived IPA was also shown to be directly captured by hepatic macrophages, where macrophage-specific AHR signaling suppressed NF-κB/S100A8/A9-associated inflammatory and profibrotic programs (Luo et al., 2025). These studies indicate that tryptophan derivatives can act directly on tissue macrophages rather than merely altering intestinal barrier function.

The AHR axis may additionally connect tryptophan metabolism to ferroptosis. In myocardial infarction, the IL4I1-derived tryptophan metabolite indole-3-pyruvic acid promoted AHR nuclear translocation, activated NRF2, restored FTH1 and GPX4, and suppressed macrophage lipid peroxidation and ferroptosis (Dong et al., 2025). The antioxidant and iron-regulatory components of this pathway overlap with mechanisms reported in disc and endplate ferroptosis studies (Yang et al., 2021; Dou et al., 2023; Ma et al., 2024). However, an equivalent metabolite–AHR–NRF2–GPX4 pathway has not been established in disc cells. Nevertheless, whether IPA, indole-3-pyruvic acid, and indole-3-carboxaldehyde have comparable receptor specificity or bioactivity in disc cells cannot be assumed.

Urolithins, generated through microbial transformation of dietary polyphenols, provide more direct evidence in cartilage-like tissues. Urolithin A reduced IL-1β-induced Fe²^+^ accumulation and malondialdehyde production while restoring glutathione (GSH), GPX4, and ferritin in articular chondrocytes. Its protective effects were mediated through AMPK activation and suppression of mTOR/HIF-1α signaling (Wang X. et al., 2026). Urolithin B similarly inhibited inflammatory chondrocyte ferroptosis by increasing FGFR3 expression and disrupting the NCOA4–FTH1 interaction, thereby suppressing ferritinophagy and restoring iron homeostasis (Li Y. et al., 2025).

The chondrocyte findings provide a rationale for investigating urolithins in disc cells (Li Y. et al., 2025; Wang X. et al., 2026). Nevertheless, disc-specific transport constraints and cellular responses require separate evaluation (Ren et al., 2023), and efficacy in articular cartilage does not establish efficacy against disc-cell ferroptosis. Direct studies are required to determine whether urolithins reach the disc, penetrate its dense extracellular matrix, and regulate AMPK–mTOR–HIF-1α or FGFR3–NCOA4–FTH1 signaling in NP and cartilage endplate cells.

### 4.4 Candidate pro-degenerative microbial metabolites and locally generated metabolic analogues

Lactate and glutarate are discussed here as local proof-of-principle examples of metabolite-controlled cell death rather than as established gut-derived mediators. Not all microbiota-associated metabolites are protective. Imidazole propionate and TMAO have been linked to detrimental inflammatory effects in non-disc models (Li D. et al., 2025; Park et al., 2025). Succinate, by contrast, remains a computationally nominated IDD candidate in the literature reviewed here (Liu et al., 2026b), rather than an experimentally established driver of disc-cell death. Their effects in IDD remain largely untested, but studies in distant tissues illustrate mechanisms that could be relevant to the disc.

Gut microbial production of imidazole propionate was sufficient to increase its systemic and brain concentrations and induce neuroinflammation, glial activation, neuronal loss, and functional deterioration through mTORC1-dependent signaling (Park et al., 2025). Although pyroptosis and ferroptosis were not established as its principal execution pathways, the study demonstrated that a gut-derived metabolite can cross a distant tissue barrier and directly promote degenerative pathology. TMAO provides a more explicit connection to inflammatory cell death: high-choline diets or TMAO supplementation increased tissue calcification and inflammation, while TMAO directly induced macrophage pyroptosis *in vitro (*Li D. et al., 2025). Endplate transport abnormalities and macrophage accumulation are documented features of disc degeneration (Nakazawa et al., 2018; Yamamoto et al., 2022; Ren et al., 2023). These features provide a rationale for testing TMAO as a candidate modifier, but the non-disc findings do not establish a TMAO-dependent mechanism in IDD.

Succinate was included among the core metabolites computationally linked to IDD-related inflammatory and lipid pathways (Liu et al., 2026b). Yet the retrieved literature did not demonstrate a causal succinate–disc-cell-death axis. It should therefore remain a prioritized candidate for experimental validation rather than be presented as an established driver of disc degeneration.

Lactate requires separate consideration. Local lactate metabolism has been implicated in oxidative injury and inflammatory responses in experimental IDD (Xia et al., 2026; Zhang Y. et al., 2026). These studies do not establish that the relevant lactate originated from intestinal microbes; intradiscal lactate detection should therefore not be treated as evidence of a gut-derived signal. Nevertheless, lactate demonstrates how a metabolite shared by host and microbial systems can couple metabolism to inflammatory cell death. In a non-disc macrophage model, endogenous lactate promoted AARS2-dependent lactylation of NLRP3 at lysine residues 24 and 565. Lactylation is a post-translational modification involving the addition of a lactyl group to protein lysine residues. This modification enhanced ASC recruitment and facilitated inflammasome assembly and pyroptosis (Liu W. et al., 2026).

Direct IDD studies reinforce the pathological importance of lactate signaling. Metabolomic, single-cell, and lactyl-proteomic analyses identified SOD1 lactylation at lysine 123 as a critical event in disc degeneration. This modification altered SOD1 conformation, impaired its antioxidant activity, increased oxidative injury, and activated p53 signaling in NP cells (Zhang Y. et al., 2026). In another study, catalytic conversion of excess lactate into alanine reduced NLRP3 activity and oxidative stress, inhibited inflammatory amplification and NP-cell senescence, and restored the biomechanical properties of regenerated disc tissue (Xia et al., 2026). Together, these studies link lactate-related metabolism to impaired antioxidant function and inflammatory signaling in IDD (Xia et al., 2026; Zhang Y. et al., 2026). They suggest a possible connection between metabolic stress and regulated cell death, but do not establish simultaneous lactate-driven pyroptosis and ferroptosis in the same disc-cell population.

Glutarate provides a contrasting example of a locally accumulated metabolite with protective activity. In NP cells, glutarate promoted SUGCT-dependent protein glutarylation, a post-translational modification involving the addition of glutaryl groups to protein lysine residues, including those in NRF2. NRF2 glutarylation facilitated its dissociation from KEAP1 and nuclear translocation, reducing iron overload and lipid peroxidation and protecting against TNF-α-induced ferroptotic injury (Yu et al., 2026). As with lactate, the study did not establish a gut microbial origin for disc glutarate. It nevertheless demonstrates that metabolite-driven post-translational modification can determine the ferroptotic threshold of disc cells.

### 4.5 Convergent checkpoints linking pyroptosis and ferroptosis

The relationship between pyroptosis and ferroptosis in IDD is unlikely to be a simple linear sequence. Rather, microbial and host metabolites may converge on shared mitochondrial, redox, autophagic, and inflammatory checkpoints that alter susceptibility to both death programs. Candidate points of convergence include mitochondrial quality control (Ma et al., 2022), NOX4-associated inflammatory and ferroptotic signaling (Zhang F. et al., 2025), ferritinophagy (Ma et al., 2024; Wang W. et al., 2026), and metabolite-dependent regulation of NRF2 (Yu et al., 2026). Their integration into a single gut-derived regulatory pathway remains to be established.

Mitochondrial dysfunction provides the most direct connection. In disc-cell models, impaired mitochondrial quality control is associated with increased mitochondrial ROS and inflammasome-related injury (Ma et al., 2022; Liang et al., 2026; Song et al., 2026). Mitochondrial DNA release and downstream STING signaling have also been implicated in experimental IDD (Xu et al., 2026). Increased PTPN22 protein expression was reported in degenerative human and rat NP cells. In rat experimental systems, *Ptpn22* silencing inhibited PI3K/AKT/mTOR signaling, enhanced mitophagy, and reduced mitochondrial dysfunction, pyroptotic injury, and matrix degradation (Liang et al., 2026). Calreticulin similarly promoted PINK1/Parkin-dependent clearance of damaged mitochondria, reduced ROS accumulation and NLRP3 activation, and preserved ECM integrity (Song et al., 2026). These findings position mitophagy as a major determinant of the pyroptotic threshold.

Oxidative stress can also contribute to ferroptotic injury when lipid peroxide accumulation exceeds cellular antioxidant capacity (Yang et al., 2014; Yang et al., 2021). This does not establish that the ROS driving inflammasome activation and ferroptosis originate from an identical intracellular pool. Direct evidence of inflammatory–ferroptotic coupling was provided by a study showing that NOX4 increased ROS, reduced mitochondrial membrane potential and GPX4, increased ACSL4, and promoted ferroptosis in NP cells. NOX4 also interacted with LRRC8A and enhanced NLRP3 activation and inflammatory cytokine secretion (Zhang F. et al., 2025). This NOX4–LRRC8A–NLRP3 circuit is among the clearest demonstrations that inflammasome signaling and ferroptotic injury can be integrated within the same degenerative disc environment.

Ferritinophagy represents another convergence point. NCOA4 acts as a cargo receptor for autophagic ferritin degradation and iron mobilization (Mancias et al., 2014). In cartilage endplate cells, excessive NCOA4-associated ferritinophagy has been linked to iron accumulation and ferroptotic injury (Ma et al., 2024). Under mechanical and oxidative stress, PIEZO1-dependent Ca²^+^ influx and YAP1 nuclear translocation increased NCOA4-mediated ferritinophagy in cartilage endplate cells. In addition to expanding the labile iron pool, ferritinophagy destabilized mitochondrial DNA and promoted the generation and release of Z-form mitochondrial DNA, which activated ZBP1 and NF-κB-dependent inflammation (Wang W. et al., 2026). Thus, ferritinophagy can connect iron mobilization to innate immune signaling rather than serving only as a ferroptotic iron-supply mechanism.

NRF2-centered antioxidant defense may counterbalance both pathways. Glutarate-induced NRF2 glutarylation inhibited iron accumulation and lipid peroxidation in NP cells (Yu et al., 2026), whereas tryptophan-derived indole-3-pyruvic acid activated an AHR–NRF2 pathway that preserved FTH1 and GPX4 and reduced macrophage ferroptosis in another tissue context (Dong et al., 2025). In parallel, suppression of mitochondrial ROS through mitophagy reduces NLRP3 activation and pyroptosis (Liang et al., 2026; Song et al., 2026). These findings support a model in which NRF2 activity and mitochondrial quality control collectively determine whether metabolic stress is resolved or progresses toward inflammatory and ferroptotic death.

Metabolite effects are therefore unlikely to be uniformly protective or harmful. Butyrate can suppress macrophage pyroptosis and inflammatory ferroptosis in some settings (Hua et al., 2025; Chen et al., 2026), yet induce NCOA4-dependent ferroptosis in metabolically distinct cancer cells (Liu L. et al., 2025). LCA-associated interventions have been reported to suppress NLRP3-related inflammatory signaling (Xu et al., 2025; Zhong et al., 2026), whereas taurodeoxycholic acid reduced caspase-11–GSDMD-associated responses in a liver-injury model (Ruan et al., 2026).Other bile acid species promoted mitochondrial injury and STING-associated inflammatory signaling in cholangiocytes (Fan et al., 2026). Lactate can promote NLRP3 lactylation and impair SOD1 activity (Liu W. et al., 2026; Zhang Y. et al., 2026), whereas its metabolic removal suppresses inflammation and oxidative stress (Xia et al., 2026). These apparently discordant findings emphasize that metabolite identity, concentration, exposure duration, cellular receptor repertoire, and disease stage must all be considered.

Collectively, these findings motivate a working hypothesis in which microbial and host metabolites may modify disc-cell susceptibility to inflammatory and iron-dependent injury. Local hypoxia, acidity, mechanical stress, mitochondrial dysfunction, and iron handling could influence these responses. However, neither a complete gut-derived causal pathway nor a combined pyroptotic–ferroptotic phenotype within the same disc-cell population has been consistently demonstrated. Changes in inflammasome components, antioxidant proteins, or lipid peroxidation require functional interpretation, and even GSDMD pore formation may be reversible (Mishima et al., 2025; Otchere et al., 2025). Future studies should combine pathway-specific genetic interventions, validated pharmacological rescue, dynamic lipid and iron measurements, and spatially resolved metabolomics to establish whether microbial metabolites causally control the pyroptosis–ferroptosis interface in IDD. The principal molecular checkpoints coupling pyroptosis and ferroptosis in IDD are illustrated in **Figure 3**.

**Figure 3:**
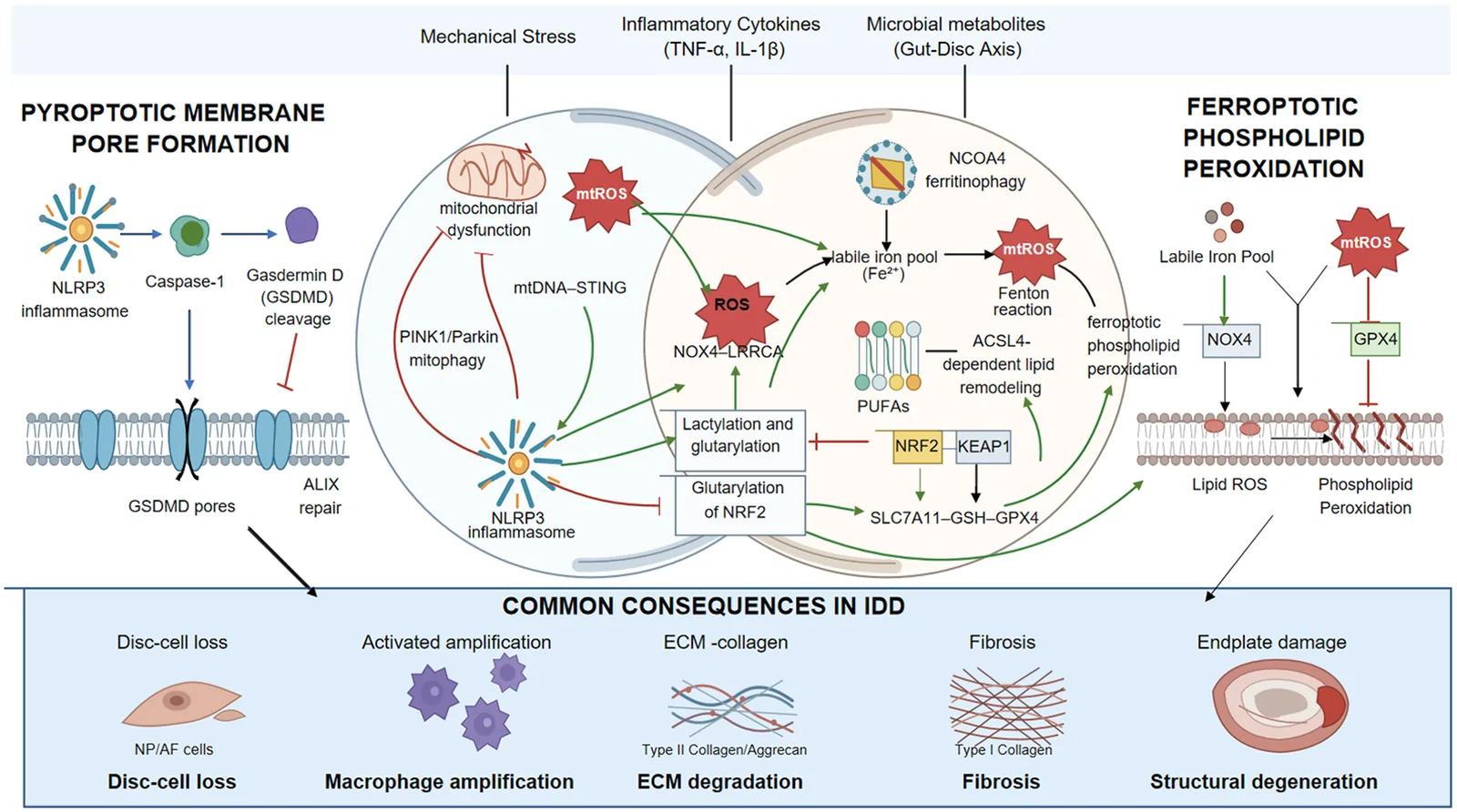
Convergent molecular checkpoints coupling pyroptosis and ferroptosis in intervertebral disc degeneration. Mechanical stress, inflammatory cytokines, and microbial metabolites may converge on mitochondrial, inflammatory, iron-regulatory, and lipid-metabolic pathways in disc cells. In the pyroptotic arm, NLRP3 inflammasome activation promotes caspase-1-dependent GSDMD cleavage and membrane-pore formation. GSDMD pores may be counteracted by ALIX-associated membrane-repair mechanisms, whereas persistent pore formation promotes inflammatory cell injury and cytokine release. Mitochondrial dysfunction and impaired PINK1/Parkin-mediated mitophagy increase mitochondrial reactive oxygen species and mitochondrial DNA–STING signaling, thereby enhancing NLRP3 activation. In the ferroptotic arm, NCOA4-mediated ferritinophagy expands the labile Fe²^+^ pool, promotes Fenton chemistry, and cooperates with ACSL4-dependent polyunsaturated phospholipid remodeling to drive lipid peroxidation. NOX4–LRRC8A signaling further contributes to reactive oxygen species generation, whereas NRF2–KEAP1 signaling and the SLC7A11–GSH–GPX4 axis oppose ferroptotic injury. Metabolite-dependent lactylation and glutarylation may modify NLRP3 and NRF2 activity, respectively, providing additional links between cellular metabolism and regulated cell death. Interactions among pyroptotic membrane injury, iron-dependent phospholipid peroxidation, and macrophage-mediated inflammation contribute to disc-cell loss, macrophage amplification, extracellular-matrix degradation, fibrosis, endplate damage, and structural degeneration. ACSL4, acyl-CoA synthetase long-chain family member 4; AF, annulus fibrosus; ALIX, ALG-2-interacting protein X; ECM, extracellular matrix; GPX4, glutathione peroxidase 4; GSDMD, gasdermin D; GSH, glutathione; IDD, intervertebral disc degeneration; IL, interleukin; KEAP1, Kelch-like ECH-associated protein 1; LIP, labile iron pool; LRRC8A, leucine-rich repeat-containing protein 8A; mtDNA, mitochondrial DNA; mtROS, mitochondrial reactive oxygen species; NLRP3, NOD-like receptor family pyrin domain-containing 3; NOX4, NADPH oxidase 4; NP, nucleus pulposus; NRF2, nuclear factor erythroid 2-related factor 2; PUFAs, polyunsaturated fatty acids; SLC7A11, solute carrier family 7 member 11; STING, stimulator of interferon genes; TNF-α, tumor necrosis factor-α. Created in BioRender. DONG, Y. (2026). https://BioRender.com/k7tse8t.

## 5 Targeting the gut–disc axis: therapeutic strategies and translational opportunities

Recognition of the gut–disc axis creates a therapeutic framework that extends beyond direct suppression of intradiscal inflammation. In principle, intervention can be implemented at several levels: remodeling the intestinal microbial ecosystem, replacing beneficial microbial products or metabolites, blocking downstream pyroptotic and ferroptotic machinery, restoring mitochondrial and immunometabolic homeostasis, and improving the delivery of therapeutic cargo to the avascular intervertebral disc. These approaches are not mutually exclusive. Indeed, the most effective strategy may require simultaneous correction of systemic microbial dysfunction and local interruption of self-sustaining inflammatory and oxidative cascades.

The current evidence base, however, remains markedly unbalanced. The reviewed literature includes direct microbiota-related interventions, such as bacterial EV administration and microbiota transfer (Ni et al., 2024; Guan et al., 2026), alongside a broader range of studies targeting local cell-death pathways and responsive delivery systems (Wang Y. et al., 2025; Wu T. et al., 2025; Zhang Y. et al., 2025; Xu et al., 2026). These categories should be distinguished because downstream efficacy does not itself validate a microbial initiating mechanism. Consequently, therapeutic discussion should distinguish interventions that directly manipulate the gut–disc axis from those that target local pathways predicted to lie downstream of microbial signaling.

### 5.1 Microbiome-directed interventions

Dietary modification, probiotics, prebiotics, FMT, and precision microbiome engineering have been investigated across microbiome-associated diseases and proposed as future strategies for musculoskeletal degeneration (Zhang YW. et al., 2026). Experimental studies in other disease settings support potential effects on intestinal barrier function, endotoxin exposure, and inflammatory signaling (Hua et al., 2025). Clinical exploration has also begun in patients with chronic low back pain and Modic changes, including a completed probiotic trial and a published Bifidobacterium trial protocol (Jensen et al., 2019; Nian et al., 2025). These studies establish clinical feasibility and research activity, but not prevention or reversal of structural IDD. Future trials should distinguish symptomatic improvement from disease modification and relate clinical outcomes to microbial changes, metabolite exposure, and longitudinal imaging.

The most compelling direct evidence currently comes from Akkermansia muciniphila. Mendelian randomization and clinical cohort analyses identified an inverse association between fecal A. muciniphila abundance and IDD risk and severity (Guan et al., 2026). In microbiota-depleted mice, administration of A. muciniphila attenuated degeneration, whereas pharmacological blockade of extracellular vesicle (EV) secretion abolished this protection. Purified A. muciniphila-derived EVs reproduced the therapeutic effect in natural ageing, needle-puncture, and bipedal-standing models (Guan et al., 2026). Proteomic and functional analyses further identified the EV-enriched effector protein B2UKX5, which regulated collagen synthesis, extracellular matrix remodeling, and chromatin-silencing pathways. Circulating and intradiscal levels of A. muciniphila EVs and B2UKX5 were also negatively correlated with disease severity in clinical samples (Guan et al., 2026). This study therefore moves beyond microbiome association and establishes a strain–EV–effector protein–disc phenotype axis with experimental evidence of causality.

FMT-related findings offer additional support but require careful interpretation. In a rat IDD model, *Mettl3* silencing reduced *Tlr2* mRNA stability, NP-cell senescence, and SASP-associated factors while altering gut microbial composition. Transfer of fecal microbiota from these donor rats modified pathological phenotypes in recipient IDD rats (Ni et al., 2024). These results demonstrate that a transferable microbial community can participate in the host phenotype. However, because the donor microbiota was generated through host gene manipulation, the study does not establish nonspecific FMT as an effective treatment. It instead suggests a reciprocal relationship in which host epigenetic regulation reshapes the microbiota, which subsequently feeds back on disc pathology.

Animal and human multi-omics studies provide a rationale for intervention but not proof of therapeutic efficacy. IDD rabbits exhibited altered gut microbial diversity and extensive changes in fecal metabolites, including pathways related to tyrosine metabolism, ascorbate metabolism, coenzyme A biosynthesis, and nucleotide metabolism (Cheng et al., 2024). Human IDD cohorts likewise showed altered abundances of Proteobacteria and Fusobacteriota and enrichment of lipid-related metabolites associated with oxidative stress and tissue degradation (Liu J. et al., 2025). These profiles may identify microbial functions suitable for restoration, but the presence of dysbiosis alone does not indicate which organism should be supplemented, removed, or metabolically redirected.

An endocrine component may also be therapeutically relevant. Glucagon-like peptide-1 (GLP-1) is regulated partly by the gut microbiota and has been proposed as a mediator connecting microbial homeostasis with inflammation, oxidative stress, regulated cell death, and tissue metabolism in degenerative musculoskeletal diseases (Yang et al., 2026). Strategies that enhance endogenous GLP-1 secretion or activate its receptor could therefore provide an indirect route for modifying the gut–disc axis. However, direct evidence that GLP-1-based treatment modifies human disc degeneration independently of its metabolic and weight-reducing effects remains insufficient.

Translation of microbiome-directed therapy will require greater precision than empirical probiotic supplementation. Human studies demonstrate substantial interindividual differences in mucosal colonization following probiotic administration (Zmora et al., 2018). FMT also requires careful donor screening, as transmission of drug-resistant bacteria has caused bloodstream infection in recipients (Defilipp et al., 2019). Defined microbial products may offer opportunities for more specific mechanistic testing, as illustrated by the A. muciniphila–EV–B2UKX5 study (Guan et al., 2026), but their comparative safety and clinical efficacy remain to be established.

### 5.2 Metabolite- and postbiotic-based therapies

Defined microbial metabolites and effectors warrant investigation as alternatives to live-microorganism administration. Under the International Scientific Association for Probiotics and Prebiotics (ISAPP) consensus, postbiotics are preparations containing inanimate microorganisms and/or their components that confer a health benefit on the host. Purified microbial metabolites alone do not meet this definition (Salminen et al., 2021). Network pharmacology and molecular docking have identified acetate, propionate, butyrate, succinate, trimethylamine N-oxide, and 3-indolepropionic acid as candidate regulators of IDD-associated targets, with IL-6, TLR4, CXCL8, and JUN emerging as highly connected nodes (Liu et al., 2026b). These predictions provide a rational shortlist for experimental screening but do not demonstrate pharmacokinetic exposure, receptor engagement, or efficacy in the disc.

Bacterial EVs provide a candidate vehicle for microbial effector delivery. In the A. muciniphila study, EV-associated B2UKX5 contributed to protective effects in experimental IDD, providing evidence for a defined bacterial EV–effector mechanism (Guan et al., 2026). The ability of A. muciniphila-derived EVs and recombinant B2UKX5 to reach disc tissues and attenuate degeneration supports the feasibility of using bacterial products as systemically administered disease-modifying agents (Guan et al., 2026). Critical unanswered questions include EV biodistribution, batch reproducibility, immune recognition, long-term toxicity, and whether therapeutic EVs preferentially accumulate in degenerated rather than healthy discs.

Studies of local disc metabolism also illustrate how metabolite-based treatment could be designed, although the microbial origin of these metabolites has not been established. Glutarate accumulated in degenerated discs but exerted a compensatory protective effect. Mechanistically, it promoted SUGCT-dependent protein glutarylation, modified NRF2, facilitated NRF2 dissociation from KEAP1 and nuclear translocation, and reduced iron overload and lipid peroxidation in NP cells (Yu et al., 2026). This finding indicates that metabolites can control ferroptotic susceptibility through post-translational modification rather than solely through conventional receptor signaling.

Lactate-targeted strategies provide another example. Experimental IDD studies associate altered lactate metabolism with oxidative injury, inflammatory signaling, and NP-cell senescence (Lu et al., 2025; Xia et al., 2026; Zhang Y. et al., 2026). An orthogonal tandem catalytic system that converted excess lactate into alanine reduced NLRP3 activity, oxidative injury, and cellular senescence and improved structural and biomechanical disc repair (Xia et al., 2026). Cryptotanshinone similarly attenuated lactate-induced NP-cell injury by modulating the STAT3/SIRT3 axis, thereby reducing oxidative stress, senescence, apoptosis, and extracellular matrix degradation (Lu et al., 2025). These studies do not establish lactate as a gut-derived mediator; rather, they show that local metabolite reprogramming can interrupt downstream pathways shared with microbial-metabolite signaling.

Several challenges must be addressed before metabolite therapy can become clinically credible. First, intradiscal exposure requires direct measurement because CEP transport is altered by degeneration (Ren et al., 2023). Second, metabolite effects cannot be generalized across tissues, as illustrated by the contrasting ferroptosis-related actions of butyrate in inflammatory and cancer models (Liu L. et al., 2025; Chen et al., 2026). Future studies should compare systemic exposure, tissue distribution, efficacy, and toxicity when evaluating unmodified metabolites, selective analogues, and targeted carriers.

### 5.3 Targeting pyroptosis and ferroptosis downstream of microbial signaling

When correction of gut dysbiosis or metabolite imbalance is not feasible, the downstream regulated-cell-death machinery provides an alternative therapeutic entry point. This approach could be evaluated in disease settings where local inflammatory and oxidative mechanisms persist after the initiating stimulus has diminished; however, such a patient subgroup has not yet been clinically defined.

Multiple studies support targeting the NLRP3–caspase-1–gasdermin axis. PTPN22 was upregulated in degenerative human and rat NP cells, and its knockdown enhanced mitophagy, improved mitochondrial function, reduced pyroptosis and matrix degradation, and attenuated degeneration through inhibition of PI3K/AKT/mTOR signaling (Liang et al., 2026). The natural compound (Z)-ligustilide stabilized ATG5 protein and promoted its interaction with NLRP3, thereby suppressing NLRP3–caspase-1–GSDMD signaling, inflammatory cytokine release, and matrix catabolism in NP cells and rat discs (Wang J. et al., 2025). In rat experimental models, *Creg1* overexpression restored PINK1/parkin-dependent mitophagy, reduced mitochondrial dysfunction and NLRP3 activation, and delayed disc degeneration (Zhang Y. et al., 2025).

EV-based interventions can deliver pyroptosis-regulating nucleic acids. Human adipose-derived stem-cell exosomes transferred miR-155-5p to NP cells, targeted TGFβR2, promoted autophagy, and reduced LPS-induced pyroptosis and matrix degradation (Chen et al., 2023). These results support cell-free delivery of regulatory RNAs but also demonstrate that the biological effect depends on defined cargo rather than on an assumed generic benefit of stem-cell EVs.

Ferroptosis-targeted strategies are similarly diverse. Duhuo Jisheng decoction-conditioned bone-marrow mesenchymal stem cells produced exosomes enriched in miR-19b-3p. These EVs targeted ACSL4, inhibited NP-cell ferroptosis, improved mitochondrial function and matrix metabolism, and ameliorated degeneration after intradiscal administration in rats (Feng et al., 2025). Deferoxamine reduced iron-dependent lipid injury and cartilage endplate degeneration by activating HIF-1α/BNIP3-mediated mitophagy, restoring GSH and GPX4, and decreasing malondialdehyde (Wang H. et al., 2025).

FBXO2 provides an example of coordinated regulation of mitochondrial quality and iron-dependent death. FBXO2 protein abundance was reduced in degenerated human NP tissues. Functional experiments linked increased FBXO2 expression to enhanced PINK1/parkin-mediated mitophagy and K27-linked polyubiquitination and proteasomal degradation of LCN2. In rats, delivery of an *Fbxo2* expression construct attenuated experimental disc degeneration (Wu T. et al., 2025). Melatonin also attenuated macrophage-conditioned-medium-induced NP-cell ferroptosis by reducing intracellular iron overload and restoring GPX4 and SLC7A11 while suppressing ACSL4 and LPCAT3 (Dou et al., 2023).

Despite these encouraging findings, several methodological considerations are essential. Changes in individual inflammasome or ferroptosis-associated markers do not establish execution of the corresponding death program. Validation should integrate complementary functional assays with genetic or pharmacological rescue (Mishima et al., 2025; Otchere et al., 2025). The comparative safety of local, stage-restricted, and continuous systemic inhibition requires direct evaluation rather than being inferred from efficacy in short-term animal models.

### 5.4 Restoring mitochondrial quality control and immunometabolic homeostasis

Targeting mitochondrial quality control may influence several degenerative processes. Separate IDD studies associate mitophagy-related interventions with reduced pyroptotic injury (Zhang Y. et al., 2025; Liang et al., 2026) or ferroptotic injury (Wang H. et al., 2025; Wu T. et al., 2025). Whether this approach outperforms selective inhibition of individual death pathways requires direct comparison.

Mitophagy is a particularly important convergence node. PTPN22 suppression, *CREG1* overexpression, deferoxamine treatment, and FBXO2 restoration all improved disc-cell survival through pathways involving mitochondrial quality control (Wang H. et al., 2025; Wu T. et al., 2025; Zhang Y. et al., 2025; Liang et al., 2026). These interventions simultaneously reduced mitochondrial ROS, inflammasome activation, lipid peroxidation, or matrix catabolism. The convergence of these phenotypes suggests that enhancing mitochondrial clearance may interrupt several degenerative pathways rather than merely suppress a single terminal death program.

The consequences of autophagy depend on the cargo and biological context. Mitophagy-related interventions have reduced oxidative injury in experimental IDD (Wang H. et al., 2025; Wu T. et al., 2025; Liang et al., 2026), whereas NCOA4-mediated ferritin degradation mobilizes iron (Mancias et al., 2014) and can contribute to ferroptotic injury in cartilage endplate cells (Ma et al., 2024). Therapeutic strategies should therefore distinguish mitochondrial quality control from excessive ferritinophagy.

Immunometabolic reprogramming is equally relevant. Macrophage-derived cytokines and ROS can induce ferroptosis in disc cells, as illustrated by the protective effect of melatonin against LPS-stimulated macrophage-conditioned medium (Dou et al., 2023). Local lactate accumulation also reinforces oxidative injury and inflammatory signaling, whereas catalytic lactate removal or STAT3/SIRT3 modulation restores metabolic balance (Lu et al., 2025; Xia et al., 2026). These findings justify testing combinations that address macrophage-associated injury, local metabolite accumulation, and mitochondrial dysfunction (Dou et al., 2023; Lu et al., 2025; Xia et al., 2026). Their benefit relative to single-cytokine blockade remains to be established.

### 5.5 Disc-targeted and responsive delivery systems

Disc anatomy and degeneration-associated changes in CEP diffusivity impose constraints that should be considered when developing systemic delivery strategies (Sun et al., 2020; Ren et al., 2023). Local needle-based access also requires careful evaluation: a long-term matched cohort study associated discography with accelerated degenerative changes, although these findings cannot be directly generalized to all intradiscal therapeutic procedures (Carragee et al., 2009). In addition, discitis and spondylodiscitis, although uncommon, have been reported after intradiscal biologic procedures (Jerome et al., 2021). Advanced biomaterials have therefore been designed to improve retention, release therapeutic cargo in response to pathological stimuli, and provide mechanical support.

A ROS-responsive nanozyme-functionalized hydrogel was developed to interrupt the ROS–ferroptosis–inflammation cycle. The system incorporated NP-cell-membrane-coated black phosphorus–cerium oxide nanozymes that provided sustained ROS scavenging through a cerium redox cycle. It also reduced ELAVL1 (HuR)-dependent stabilization of the transcript encoding IL-6 and attenuated IL-6/STAT3-associated inflammatory and ferroptotic signaling. Therapeutic effects were evaluated in a rat puncture-induced IDD model (Wang Y. et al., 2025).

Responsive delivery can also target immune amplification. A dual-network hydrogel enabled ROS-triggered release of wedelolactone and disrupted the NF-κB–LCN2 feedback loop between macrophages and AF cells. Treatment restored extracellular matrix programs, reduced inflammatory signaling, preserved MRI signal and disc height, and improved segmental biomechanics in a rat AF-defect model (Wu Z. et al., 2026). Similarly, ROS-responsive microspheres carrying shTMEM2-loaded liposomes reduced mitochondrial permeability transition, mtDNA release, STING–TBK1–NF-κB signaling, and NLRP3-mediated pyroptosis after a single intradiscal administration (Xu et al., 2026).

Hydrogels can additionally carry genome-editing or RNA-modifying cargo. A spherical GelMA–HAMA system delivered an APET×2 polypeptide and a single-guide RNA (sgRNA) targeting *Nudt21*, the mouse gene encoding the cleavage factor protein CFIm25. This intervention reduced p38/NF-κB-associated inflammation, enhanced NP-cell proliferation and migration, restored type II collagen and aggrecan, and delayed degeneration in mice (Yu et al., 2024). A nanoCRISPR-assisted biomimetic AF patch combined aligned structural support with CRISPR-mediated VEGF suppression, thereby inhibiting endothelial-to-mesenchymal transition, pathological angiogenesis, and inflammatory exposure following AF injury (Huang et al., 2025).

EV–hydrogel combinations provide sustained immunomodulation. A silk-fibroin hydrogel loaded with engineered bone-marrow mesenchymal stem-cell EVs inhibited M1 macrophage polarization, promoted reparative markers, reduced inflammatory infiltration, and improved disc histology. S100B was identified as a key EV cargo responsible for suppressing inflammatory macrophage activation (Liu Q. et al., 2025).

Noninvasive systemic targeting is beginning to emerge. An orally administered biomimetic platform used yeast microcapsules and ROS-responsive nanoparticles to deliver celastrol. Macrophage “hitchhiking” facilitated transport toward inflamed discs, where ROS triggered drug release. The system simultaneously inhibited inflammasome activation, reprogrammed macrophage polarization, and modified lipid metabolism (Ding et al., 2026). Although this platform did not directly manipulate gut microbes, it demonstrates that oral therapy can exploit intestinal uptake and immune-cell trafficking to reach the disc.

Single-cell-informed material design may further improve selectivity. A self-propelled nanocarrier was designed after single-cell analysis identified a fibrotic barrier and STING-mediated NP-cell pyroptosis. Hydrogen peroxide-driven propulsion enhanced penetration into degenerative tissue, while siRNA-mediated STING inhibition reduced inflammation and pyroptosis without substantial accumulation in healthy discs (Zhou H. et al., 2026). This mechanism–target–material integration represents a possible route toward phenotype-specific treatment.

Important translational barriers remain. Most delivery systems have been evaluated in rodent puncture models, which do not fully reproduce the dimensions, diffusion distances, mechanical loading, chronicity, or endplate transport of human lumbar discs. Long-term degradation, material fatigue, immunogenicity, off-target distribution, sterility, manufacturing reproducibility, and compatibility with future surgery require systematic evaluation. Gene-editing systems additionally raise concerns regarding persistent off-target effects. Large-animal studies and clinically realistic administration procedures are therefore essential.

### 5.6 Precision stratification and biomarker-guided intervention

Molecular classification of human disc tissues identifies biologically distinct IDD subtypes (Jin et al., 2025), supporting investigation of whether treatment response differs across disease phenotypes. Precision intervention will require integration of microbial, metabolic, molecular, and imaging phenotypes.

Transcriptomic classification has begun to define biologically distinct disease states. Analysis of 122 NP tissues identified four lumbar disc degeneration subtypes characterized by collagenesis, ossification, low chondrogenesis, and fibrogenesis. The fibrogenic subtype was strongly associated with macrophage-derived TNF-α, whereas other subtypes were linked to mechanosensing, progenitor-cell differentiation, or impaired hypoxic adaptation. A random-forest model using clinical variables achieved an area under the receiver-operating-characteristic curve of 0.9312 for subtype classification (Jin et al., 2025). These findings indicate that an immunomodulatory or microbiome-based strategy may be most relevant to an inflammatory or fibrogenic subtype rather than to every degenerative disc.

Circulating biomarkers could complement tissue classification. Plasma proteomic profiling identified COL6A3, REG1B, ATF5, CAP1, MAGEA4, and LILRB3 as a six-protein panel for distinguishing different IDD grades, with a combined validation AUC of 0.87 (Zhou Z. et al., 2026). Although these proteins are not specific to the gut–disc axis, they could be incorporated into multimodal models to monitor systemic inflammation, matrix remodeling, or therapeutic response.

Imaging biomarkers offer a noninvasive measure of tissue phenotype. In a cohort of 1,397 individuals, MRI radiomics extracted from deep-learning disc segmentations achieved a balanced accuracy of 76.7% and a Cohen κ of 0.70 for Pfirrmann-grade classification, outperforming conventional height and signal-intensity indices (Mcsweeney et al., 2025). Radiomic features may eventually identify discs with inflammatory, fibrotic, oxidative, or endplate-dominant characteristics that are not captured by conventional grading.

Microbiome signatures could add a further layer. In patients with Modic changes, shotgun metagenomics identified reduced microbial diversity and discriminative taxa, including unclassified Parabacteroides and Bacteroides uniformis, with AUC values of 0.895 and 0.889, respectively. Quorum-sensing and glycerolipid-metabolism pathways correlated with C-reactive protein and Pfirrmann grade (Lan et al., 2025). The inverse correlation between A. muciniphila, its EV-associated B2UKX5, and IVDD severity further supports the possibility of using microbial or circulating bacterial-product markers for stratification (Guan et al., 2026).

A future precision platform could integrate stool metagenomics, circulating microbial metabolites and EVs, intestinal barrier markers, plasma inflammatory and ferroptotic proteins, MRI radiomics, and clinical covariates. Such integration might identify endotoxemia-dominant, SCFA-deficient, inflammatory-pyroptotic, iron-overload-ferroptotic, fibrotic, or mixed phenotypes. However, these proposed endotypes, defined as disease subgroups distinguished by underlying biological mechanisms, remain conceptual and have not been validated for clinical treatment selection. Existing biomarker studies differ in sample size, study design, and validation strategy (Jin et al., 2025; Lan et al., 2025; Mcsweeney et al., 2025; Zhou Z. et al., 2026). Their ability to classify degeneration should be distinguished from prospective validation of treatment-response prediction, which remains necessary before these markers can guide therapy. Prospective longitudinal cohorts and biomarker-enriched clinical trials will be required to determine whether these signatures can guide therapy rather than merely classify disease.

In summary, targeting the gut–disc axis offers multiple therapeutic opportunities, ranging from defined microbial strains and bacterial EVs to metabolite modulation, regulated-cell-death inhibition, mitochondrial restoration, and responsive local delivery. The field is progressing from associative microbiome studies toward molecularly defined interventions, exemplified by the A. muciniphila–EV–B2UKX5 axis. Most mechanistically defined strategies remain preclinical (Wang Y. et al., 2025; Wu Z. et al., 2026; Xu et al., 2026). Although probiotic interventions have entered clinical investigation in Modic-associated low back pain, convincing evidence of structural disease modification in human IDD remains lacking (Jensen et al., 2019; Nian et al., 2025). Translation will depend on establishing causal microbial functions, defining tissue pharmacokinetics, matching interventions to molecular disease subtypes, and developing delivery systems compatible with the unique biological and mechanical constraints of the human disc.

## 6 Translational challenges and unresolved questions

Despite rapidly accumulating evidence supporting communication between the gut microbiome and the intervertebral disc, translation of the gut–disc axis from an attractive biological concept into a clinically actionable framework remains constrained by several unresolved methodological and mechanistic problems. The central limitation is not simply a shortage of candidate microorganisms, metabolites, or signaling pathways. The evidence is distributed across distinct experimental levels: clinical studies describe microbial and metabolic associations (Rajasekaran et al., 2024; Liu J. et al., 2025; Liu et al., 2026a), disc-cell experiments investigate local death pathways (Ma et al., 2022; Ma et al., 2024), and intervention studies test selected microbial products or downstream targets in animal models (Wang Y. et al., 2025; Guan et al., 2026). These levels have not been consistently connected within a single causal framework. Few studies have connected these levels within the same experimental system.

A clinically persuasive gut–disc axis will ultimately require demonstration of a temporally ordered causal chain: a defined alteration in intestinal microbial function must precede disease progression; the corresponding microbial product must reach the systemic circulation and disc compartment at a biologically relevant concentration; a specific receptor or intracellular target must be engaged in a defined disc or immune-cell population; and pathway-specific perturbation must alter pyroptosis, ferroptosis, tissue degeneration, and clinically meaningful outcomes. Among the studies reviewed here, the complete causal sequence has not been demonstrated within a single experimental and clinical framework.

### 6.1 From association to causality

Most human evidence for the gut–disc axis remains associative. The initial pilot case–control study included 40 patients with disc degeneration and 20 healthy volunteers and identified dysbiosis, depletion of short-chain fatty acid-producing bacteria, and associations with inflammatory and imaging phenotypes (Rajasekaran et al., 2024). Subsequent fecal microbiome and metabolomic studies reported additional taxonomic and metabolic differences between patients with and without intervertebral disc degeneration (IDD) (Liu J. et al., 2025). Although these findings are biologically coherent, cross-sectional sampling cannot determine whether dysbiosis initiates degeneration, accelerates an established pathological process, or develops secondarily to disease-associated changes in diet, mobility, medication use, sleep, and metabolic health.

Surgically obtained disc tissues provide greater anatomical proximity to disease but introduce another form of selection bias. Patients undergoing discectomy or fusion usually represent symptomatic and relatively advanced disease rather than the broader population with early or asymptomatic radiographic degeneration. A recent paired fecal–disc multi-omics study identified shared microbial species and associated Phocaeicola vulgatus with LPS–TLR4–MYD88 signaling, immune-cell communication, angiogenesis, and fibrosis in degenerative discs (Liu et al., 2026a). This study substantially strengthened biological plausibility but remained based on an operative cohort and could not determine whether microbial exposure preceded structural degeneration.

Mendelian randomization offers a complementary strategy by using genetic instruments to reduce conventional environmental confounding. Analyses have reported potential causal associations between several microbial taxa and intervertebral disc disorders (Ge et al., 2025). However, another Mendelian randomization study did not support mediation through the circulating inflammatory cytokines examined (Yan et al., 2026). These findings suggest that systemic cytokinemia may not be the only or dominant intermediate, but they do not identify the responsible microbial strain, metabolite, exposure window, or disc-cell target. Interpretation of Mendelian randomization requires explicit assessment of instrument relevance, potential confounding of genetic associations, and pathways through which instruments may affect the outcome independently of the exposure (Skrivankova et al., 2021). Microbiome-specific analyses additionally require attention to the taxonomic resolution and populations represented in the underlying datasets (Ge et al., 2025; Yan et al., 2026).

The Akkermansia muciniphila–extracellular vesicle–B2UKX5 study represents an important advance because it combined genetic inference, clinical association, microbiota-depleted animals, extracellular-vesicle inhibition, recombinant protein validation, and several IDD models (Guan et al., 2026). Nevertheless, the therapeutic component remains preclinical. It has not yet been established whether supplementation with A. muciniphila, its extracellular vesicles, or B2UKX5 can safely modify disc degeneration in humans.

Future studies should move beyond isolated association and incorporate causal perturbation. Particularly informative designs would include longitudinal sampling before radiographic progression, gnotobiotic colonization with defined microbial strains, transfer of microbial communities from well-characterized donors, metabolite depletion and add-back experiments, receptor-specific genetic deletion, and rescue studies demonstrating that restoration of the proposed mediator reverses the phenotype.

### 6.2 Limited cohort size, cross-sectional design, and disease heterogeneity

The clinical microbiome studies reviewed here predominantly provide case–control or cross-sectional associations, with several cohorts having limited sample sizes (Aboushaala et al., 2024; Rajasekaran et al., 2024; Lan et al., 2025; Liu J. et al., 2025). Such studies are valuable for hypothesis generation but are vulnerable to unstable effect estimates, overfitting of microbial classifiers, center-specific dietary and geographic effects, and incomplete control of clinical covariates. Population-level research demonstrates that gut microbial variation is associated with multiple host and environmental covariates (Falony et al., 2016), emphasizing the need to distinguish disease-related signals from background variation.

Clinical heterogeneity further complicates interpretation. IDD is not a uniform disease entity. Pfirrmann grading, Modic changes, disc herniation, degenerative spondylolisthesis, endplate damage, fibrosis, and discogenic pain represent overlapping but non-equivalent phenotypes. Degenerative imaging findings are common in asymptomatic individuals and cannot alone identify a pain-generating lesion (Brinjikji et al., 2015). Molecular profiling additionally identifies distinct biological subtypes of disc degeneration (Jin et al., 2025), indicating that imaging and molecular classification capture different aspects of disease.

Transcriptomic analysis of 122 nucleus pulposus specimens identified four molecularly distinct lumbar disc degeneration subtypes characterized by collagenesis, ossification, reduced chondrogenesis, and fibrogenesis, with the fibrogenic subtype showing a prominent macrophage–TNF-α signature (Jin et al., 2025). This heterogeneity implies that a microbiome-directed intervention may be effective only in a subset of patients with a dominant immune–metabolic phenotype. Pooling mechanistically distinct patients could therefore obscure genuine microbial associations or produce signatures that are difficult to reproduce.

Another statistical issue arises when multiple discs are obtained from the same patient. These specimens are biologically correlated and should not be treated as independent observations. Future studies should employ multilevel models that account for patient-level clustering, spinal level, disease stage, and coexisting endplate or facet pathology.

Longitudinal cohorts are essential to resolve temporality. Repeated stool, plasma, imaging, pain, and functional assessments would help determine whether microbial or metabolic changes precede MRI progression, track with disease activity, or emerge only after clinical deterioration. Prospective designs should also distinguish incident degeneration from progression of established disease.

### 6.3 Stool microbiota is an incomplete proxy for intestinal function

Stool sampling is suitable for repeated microbiome assessment, but paired sampling studies demonstrate that fecal and mucosa-associated communities can differ according to tissue compartment and disease context (Hou Y. et al., 2022; Mukhopadhya et al., 2022; Dutta et al., 2025). Stool profiles should therefore not be assumed to represent all intestinal microbial niches.

Paired analyses have demonstrated disease- and niche-dependent discordance. In irritable bowel syndrome, several taxa showed opposite abundance patterns in fecal and mucosal samples, and mucosal alterations were more closely aligned with clinical manifestations (Hou Y. et al., 2022). In healthy volunteers, fecal samples and rectal biopsy washes showed substantial similarity, supporting the usefulness of stool for some luminal communities; however, intact biopsy tissues displayed a distinct microbial signature with increased Proteobacteria and Acidobacteria (Mukhopadhya et al., 2022). In active Crohn’s disease, tissue and stool communities differed in composition and predicted function, and tissue microbiota showed better diagnostic discrimination than fecal microbiota (Dutta et al., 2025).

These apparently different findings are not contradictory. Rather, they indicate that the representativeness of stool depends on the biological question, intestinal region, sampling method, disease state, and microbial niche. The extent to which stool represents tissue-associated communities depends on the sampling method and biological question, as illustrated by paired fecal and mucosal studies (Dutta et al., 2025).

Taxonomic profiling does not directly quantify microbial metabolic activity. The taxonomic resolution of 16S rRNA sequencing depends on the region and sequencing approach: short variable-region reads generally provide less resolution than full-length sequencing (Johnson et al., 2019). Functional claims therefore require complementary measurements rather than inference from taxonomic abundance alone.

The same caution applies to fecal metabolomics. A metabolite detected in stool may reflect dietary intake, microbial production, host secretion, intestinal absorption, or incomplete degradation. Its fecal concentration does not necessarily indicate systemic exposure. Metabolite annotations based on database matching should be distinguished from confirmed identifications. The Metabolomics Standards Initiative recommends reporting identification confidence and using comparison with authentic standards for the highest-confidence assignments (Sumner et al., 2007). Consequently, future gut–disc studies should integrate shotgun metagenomics, metatranscriptomics, targeted metabolomics, microbial culture, and direct functional assays rather than relying on taxonomic composition alone.

### 6.4 Contamination and analytical instability in low-biomass disc samples

The proposed existence of an intervertebral disc microbiome remains one of the most methodologically sensitive areas in the field. Disc tissue contains extremely low microbial biomass relative to stool, oral, or skin samples. In low-biomass samples, contaminating DNA from extraction kits and laboratory reagents can substantially distort microbial profiles (Salter et al., 2014). Disc studies additionally require controls for contamination introduced during tissue acquisition and processing (Mengis et al., 2024; Fierer et al., 2025).

A study of 70 discs from patients with and without Modic changes attempted to reproduce previously reported disc microbiome findings under aseptic conditions with contamination controls (Mengis et al., 2024). There was limited overlap with the earlier study, and no bacterial genera were shared when equivalent bioinformatic parameters were applied. The choice of taxonomic assignment method increased the number of detected genera from 22 to 48, whereas changing the prevalence threshold reduced the number from 48 to four. Although both studies suggested enrichment of Gram-negative organisms in Modic-change discs, the lack of taxonomic replication demonstrated that bioinformatic settings, sample preparation, contamination, and true biological variability can materially alter conclusions.

Detection of Cutibacterium or other potential contaminants requires interpretation alongside sampling controls, absolute microbial burden, and contaminant-aware analysis (Fierer et al., 2025). DNA detection alone cannot establish viable colonization, metabolic activity, or intestinal origin.

Low-biomass disc microbiome studies therefore require more stringent evidence than conventional fecal surveys. At minimum, investigations should include operating-room controls, instrument swabs, extraction blanks, polymerase-chain-reaction blanks, reagent-lot controls, randomized extraction and sequencing batches, absolute bacterial quantification, and contaminant-aware bioinformatic analysis (Fierer et al., 2025). Ideally, sequencing should be complemented by culture, viability assays, fluorescence *in situ* hybridization, spatial localization, electron microscopy, or microbial RNA detection. Demonstration of gut origin would require strain-level genomic matching between fecal, blood, and disc isolates rather than genus-level overlap.

Without harmonized sampling and analytical protocols, apparent differences between disease groups may remain inseparable from technical variation.

### 6.5 Translational limitations of current animal models

Animal models are indispensable for causal manipulation, but no existing model fully reproduces the slow, heterogeneous, mechanically complex progression of human IDD. Needle-puncture models provide experimentally accessible models of structural injury, but reproduce only selected features of human IDD (Zhang C. et al., 2026). Ageing and other chronic models may be necessary when investigating slowly evolving processes such as cellular senescence and epigenetic change (Cui et al., 2026).

A recent review of IDD models concluded that injury-induced, spontaneous, mechanical, and chemical models each reproduce selected pathological features but struggle to capture the long-term progression and heterogeneity of human disease (Zhang C. et al., 2026). Another methodological review specifically advocated moving beyond acute puncture toward ageing and genetically engineered progeroid models when studying chronic epigenetic drift and cellular senescence (Cui et al., 2026).

Species and anatomical differences are equally important. Species, spinal region, disc anatomy, and loading conditions influence the translational relevance of experimental IDD models (Bonilla et al., 2025; Zhang C. et al., 2026). Young animals without metabolic comorbidities also provide an incomplete representation of older patients with complex clinical disease.

Large animals improve anatomical and biomechanical relevance but introduce cost, ethical, logistical, and throughput constraints. Sheep show similarities to humans in disc architecture, loading, cell composition, and molecular signaling and may provide a useful bridge between rodent studies and clinical translation (Bonilla et al., 2025). Goat models can also reproduce structural degeneration, immune-cell infiltration, innervation, and neuroinflammatory changes (Zhang C. et al., 2026). Nevertheless, induced large-animal degeneration remains an approximation and often lacks the systemic metabolic and microbial complexity of human disease.

Microbiome transplantation introduces an additional layer of species bias. Transfer of human ulcerative-colitis microbiota into germ-free mice produced donor-dependent bacterial engraftment but incompletely transferred fungal communities and altered microbial metabolic exchange compared with the original human ecosystem (Guggeis et al., 2025). An unrecognized Clostridioides difficile infection was also transferred from one donor. Thus, a phenotype observed after human-to-mouse fecal transfer may reflect selective engraftment, host filtering, metabolic divergence, or unintended pathogen transmission rather than faithful reconstruction of the donor microbiome.

Future models should combine age-appropriate mechanical degeneration with diet-induced metabolic dysfunction, defined microbial colonization, and clinically relevant comorbidities. Replication in large animals should precede clinical testing of systemic microbiome or metabolite interventions.

### 6.6 Uncertain tissue exposure and pharmacokinetics of microbial metabolites

A major unresolved question is whether candidate microbial metabolites reach the disc at concentrations sufficient to alter cell behavior. Fecal and plasma measurements characterize different sampling compartments and should not be treated as interchangeable exposure measures. Although microbial metabolism influences circulating metabolites (Wikoff et al., 2009), disc transport constraints require intradiscal exposure to be evaluated separately (Ren et al., 2023).

The IVD is largely avascular and depends predominantly on diffusion across the vertebral endplate for nutrient and solute exchange. Degeneration-associated changes in the CEP can reduce solute diffusivity (Crump et al., 2023; Ren et al., 2023).Whether and to what extent this limits exposure to individual microbial metabolites or systemic therapeutics requires molecule-specific investigation.

The biological origin of metabolites detected in disc cells is also uncertain. Degenerated human disc cells exhibit altered lipidomic profiles, including changes in bile acids, ceramides, fatty acids, phospholipids, and sphingomyelins (Francisco et al., 2023). However, detection of a bile acid or other metabolite in disc cells does not demonstrate microbial origin. Similarly, local lactate-related mechanisms have been demonstrated in experimental IDD (Xia et al., 2026; Zhang Y. et al., 2026), but these findings do not establish a microbial origin for intradiscal lactate.

Concentration-dependent and cell-specific effects create an additional challenge. Butyrate has been associated with suppression of macrophage pyroptosis or ferroptosis-related injury in inflammatory models (Hua et al., 2025; Chen et al., 2026), but can promote ferritinophagy and ferroptosis in colorectal cancer cells (Liu L. et al., 2025; Chen et al., 2026). Likewise, individual bile acids have shown contrasting inflammatory and mitochondrial effects across experimental settings (Xu et al., 2025; Fan et al., 2026; Ruan et al., 2026). A therapeutic concentration in intestinal immune cells may therefore be ineffective or harmful in disc cells.

Future studies should use isotope-labeled microbial substrates or metabolites to track intestinal production, systemic transport, tissue entry, and intracellular metabolism. Paired fecal, portal-vein, peripheral-blood, endplate, and disc measurements should be obtained using quantitative targeted metabolomics with authentic standards. Spatial metabolomics and receptor-engagement assays will be necessary to distinguish mere detection from biologically active tissue exposure.

### 6.7 Inconsistent identification of pyroptosis and ferroptosis

The expanding literature on regulated cell death in IDD is accompanied by substantial inconsistency in diagnostic criteria. Increased expression of an inflammasome component or ferroptosis-associated protein does not by itself establish execution of the corresponding death program; complementary functional evidence is required (Mishima et al., 2025; Otchere et al., 2025).

Pyroptosis is commonly inferred from increased NLRP3, cleaved caspase-1, IL-1β, IL-18, or GSDMD. However, inflammasome activation and cytokine maturation can occur without irreversible lytic cell death. Standardized detection should combine pathway activation with evidence of gasdermin cleavage, membrane-pore formation, membrane permeability, cellular swelling or rupture, and release of cytosolic contents. Pharmacological or genetic disruption of the responsible gasdermin or inflammatory caspase should rescue the phenotype (Yang and Xu, 2026).

The distinction between gasdermin pore formation and cell death is particularly important. GSDMD pores can be removed through an ALIX- and endosomal-sorting-complex-dependent repair mechanism. Short-duration pore formation and membrane permeability may therefore be reversible, whereas prolonged pore persistence causes extensive membrane damage and death (Otchere et al., 2025). Consequently, GSDMD-N detection alone cannot establish terminal pyroptosis.

Ferroptosis is similarly prone to overinterpretation. Changes in total ROS, malondialdehyde, Fe²^+^, GPX4, SLC7A11, ACSL4, or mitochondrial morphology are individually insufficient because these features overlap with oxidative stress, mitochondrial dysfunction, apoptosis, and other forms of cell injury. Expert recommendations emphasize the use of orthogonal assays, validated ferroptosis modulators, appropriate genetic models, and pathway-specific rescue rather than reliance on a single biomarker (Mishima et al., 2025).

Ferroptosis assessment should combine evidence of iron-dependent lipid peroxidation with validated pharmacological or genetic interventions and appropriate rescue experiments (Mishima et al., 2025). Alternative explanations for cell injury should be examined rather than relying on a single marker or mitochondrial morphology. Hyperoxidized mitochondrial PRDX3 has been proposed as a more specific tissue-compatible ferroptosis marker because conventional C11-BODIPY, MDA, and 4-HNE measurements have specificity or *in vivo* feasibility limitations (Sun et al., 2026). Nevertheless, even a promising biomarker should be interpreted within a multiparametric validation framework.

This issue is especially important at the proposed pyroptosis–ferroptosis interface. Given the limitations of marker-based death assignments (Mishima et al., 2025), simultaneous changes in NLRP3 and GPX4 should not be interpreted as proof that pyroptosis and ferroptosis occur in the same cell or that one causes the other. Spatially resolved imaging, single-cell analysis, temporal experiments, and pathway-selective rescue are required to distinguish cellular coexistence, intercellular coupling, and sequential death transitions.

### 6.8 Confounding, batch effects, and reproducibility

Gut microbiome variation is associated with multiple host and environmental covariates (Falony et al., 2016), and medication use can influence microbial composition and functional potential (Vich Vila et al., 2020; Larsson et al., 2024). These factors should be considered when interpreting microbiome–IDD associations. These variables may create confounding or effect modification rather than simple background noise.

Reverse causality is particularly plausible. Patients with chronic low back pain may reduce physical activity, alter diet and sleep, gain weight, and use NSAIDs, opioids, proton-pump inhibitors, antibiotics, metformin or other antidiabetic agents, and dietary supplements. Several of these exposures have been associated with changes in gut microbial composition, functional potential, intestinal barrier integrity, or circulating metabolites (Vich Vila et al., 2020; Cruz-Lebrón et al., 2021; Mueller et al., 2021; Larsson et al., 2024). A disease-associated microbial signature could therefore reflect treatment exposure or altered lifestyle rather than a causal driver of degeneration.

Experimental studies illustrate the magnitude of environmental effects. A high-fat diet altered both fecal and mucosal microbial communities while increasing circulating and fecal LPS and tissue IL-6 and TNF-α (Sagkan-Ozturk and Arpaci, 2022). Antibiotic exposure can leave a lasting imprint on gut microbial composition despite substantial recovery after treatment (Palleja et al., 2018). Consequently, exclusion of recent antibiotic use alone is insufficient; cumulative medication history, dietary intake, metabolic status, and physical activity should be measured systematically (Aasmets et al., 2025).

Technical variation is another source of irreproducibility. Stool collection method, time to freezing, storage temperature, freeze–thaw cycles, DNA extraction kit, amplified 16S region, sequencing platform, read depth, taxonomic database, normalization method, prevalence threshold, and batch correction can all alter results. Multi-omics studies additionally face cross-platform variability and incomplete external validation. A methodological assessment of microbiota–metabolite research highlighted the combined limitations of small samples, single-center recruitment, cross-sectional design, dietary and medication confounding, and metabolite identification without chemical-standard confirmation (Zhang TT. et al., 2026).

Future studies should use prespecified protocols, standardized metadata collection, randomized sample processing, blinded analysis, negative and positive controls, independent validation cohorts, and transparent reporting of all preprocessing decisions. Raw sequencing and metabolomic data, analytical code, and contaminant-filtering rules should be publicly available whenever possible.

### 6.9 Limited longitudinal and clinical intervention evidence

The clinical studies summarized in Section 5.1 demonstrate early investigation of microbiome-directed treatment, but they do not establish structural disease modification (Jensen et al., 2019; Nian et al., 2025). Major gaps include limited completed intervention evidence, recruitment based on pain and Modic phenotypes rather than a uniform IDD definition, and insufficient linkage between clinical outcomes and microbial or metabolic target engagement. Symptom improvement, microbiome alteration, and attenuation of disc degeneration should therefore be treated as distinct outcomes.

Prospective observational cohorts should complement intervention trials by establishing temporal relationships among microbial composition, circulating metabolites, intestinal-barrier function, imaging progression, pain, and disability. Sampling should occur at multiple time points and include asymptomatic individuals, early degeneration, progressive disease, and advanced surgical phenotypes.

Early-phase intervention studies should initially prioritize safety, pharmacokinetics, target engagement, and biological plausibility. For live biotherapeutics or bacterial extracellular vesicles, outcomes should include engraftment or circulating cargo, immune responses, antimicrobial resistance, off-target tissue distribution, and long-term safety. For metabolites, direct measurement of plasma and disc exposure is essential. For pyroptosis- or ferroptosis-targeted agents, pathway-specific biomarkers should be included rather than relying solely on MRI signal or inflammatory cytokines.

Clinical endpoints should extend beyond microbial abundance. Relevant outcomes include pain intensity, disability, analgesic consumption, work participation, MRI-based structural and quantitative biochemical measures, Modic-change evolution, systemic and local metabolite profiles, and validated regulated-cell-death markers. Imaging improvement should not automatically be equated with pain relief, and symptomatic benefit should not be assumed to indicate structural regeneration.

Patient enrichment may be necessary. Trials could selectively recruit individuals with microbial depletion, endotoxemia, a defined metabolite deficiency, an inflammatory–pyroptotic profile, an iron-overload–ferroptotic phenotype, or a combined immune–metabolic signature. Such biomarker-guided designs may be more informative than testing a uniform intervention across biologically heterogeneous IDD.

Collectively, these limitations define a minimum translational standard for the field. A candidate gut–disc mechanism should demonstrate temporal association, causal perturbability, verified metabolite or microbial-product delivery to the disc, cell-type-specific target engagement, rigorous identification of regulated cell death, and improvement in clinically meaningful outcomes. Until these criteria are met, the gut–disc axis should be regarded as a promising but incompletely validated framework rather than an established therapeutic pathway. A stepwise translational framework for validating and therapeutically targeting the gut–disc axis is proposed in **Figure 4**.

**Figure 4:**
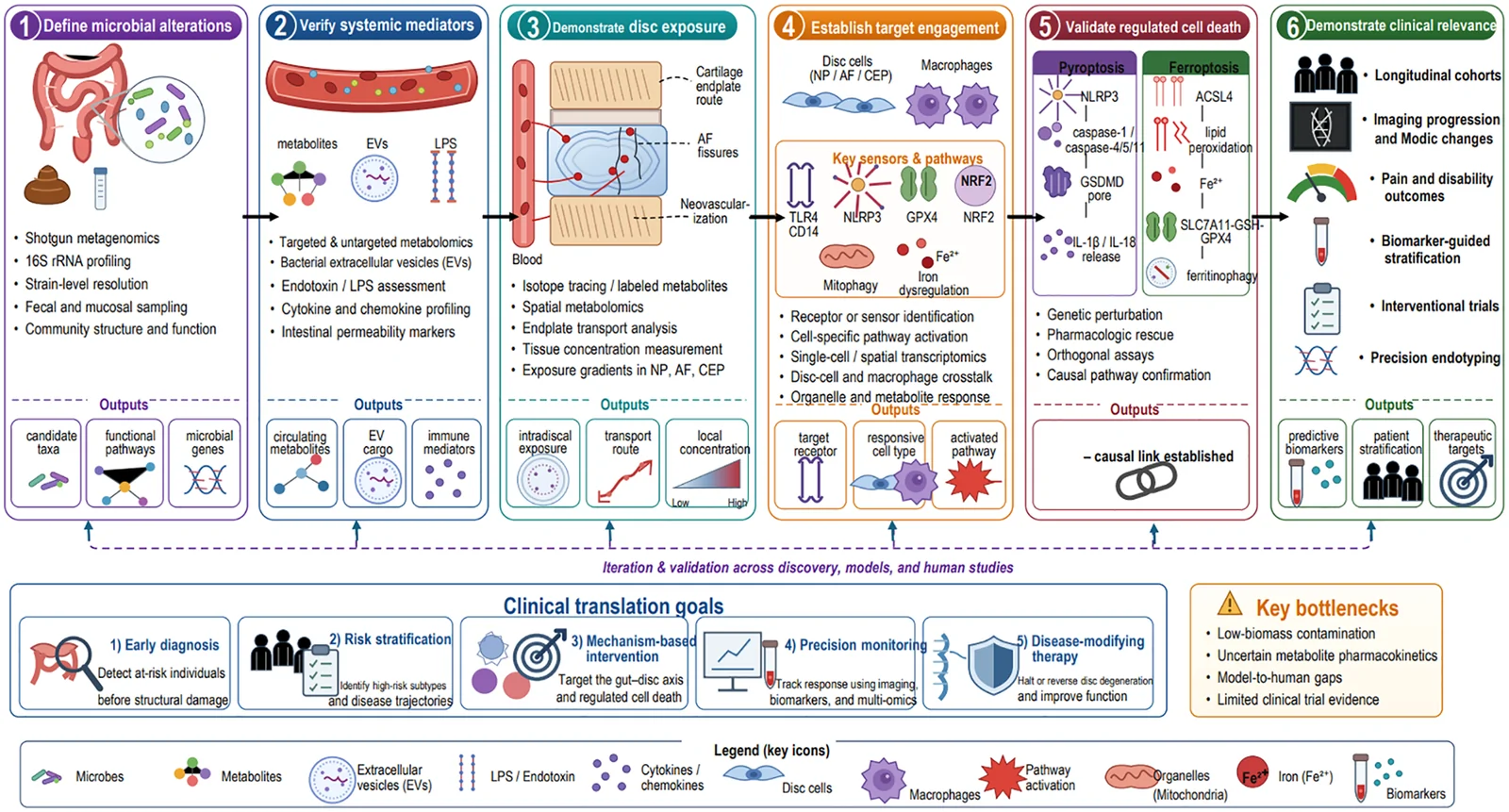
Translational roadmap for validating and targeting the gut–disc axis. A stepwise framework is proposed for progressing from microbiome association to clinically actionable gut–disc mechanisms. First, microbial alterations should be defined at taxonomic, strain, gene, and functional levels using fecal and mucosal sampling, 16S rRNA profiling, and shotgun metagenomics. Second, candidate systemic mediators should be verified through targeted and untargeted metabolomics, bacterial extracellular-vesicle characterization, endotoxin assessment, cytokine and chemokine profiling, and measurement of intestinal-barrier dysfunction. Third, intradiscal exposure should be demonstrated using isotope tracing, labeled metabolites, spatial metabolomics, cartilage-endplate transport analysis, and direct tissue-concentration measurements. Fourth, cell-type-specific target engagement should be established in nucleus pulposus, annulus fibrosus, cartilage-endplate, and immune-cell populations through receptor identification, single-cell or spatial transcriptomics, and functional pathway analysis. Fifth, pyroptosis and ferroptosis should be validated using genetic perturbation, pharmacological rescue, orthogonal assays, and pathway-specific causal experiments. Sixth, clinical relevance should be evaluated in longitudinal cohorts and interventional trials incorporating imaging progression, Modic changes, pain, disability, predictive biomarkers, patient stratification, and precision endotyping. Iterative validation across discovery studies, experimental models, and human cohorts is required to overcome low-biomass contamination, uncertain metabolite pharmacokinetics, model-to-human gaps, and limited clinical-trial evidence. AF, annulus fibrosus; CEP, cartilage endplate; EVs, extracellular vesicles; GPX4, glutathione peroxidase 4; GSDMD, gasdermin D; IDD, intervertebral disc degeneration; LPS, lipopolysaccharide; NLRP3, NOD-like receptor family pyrin domain-containing 3; NP, nucleus pulposus; NRF2, nuclear factor erythroid 2-related factor 2; SLC7A11, solute carrier family 7 member 11; TLR4, Toll-like receptor 4. Created in BioRender. DONG, Y. (2026). https://BioRender.com/slh470z.

## 7 Conclusion

The gut–disc axis expands the current understanding of IDD from a locally initiated structural disorder to a condition that may also be modified by systemic microbial, metabolic, and immune factors. Human studies associate gut microbial and metabolic alterations with disc-related disease phenotypes (Rajasekaran et al., 2024; Liu J. et al., 2025; Liu et al., 2026a), while experimental work supports a protective bacterial EV–effector mechanism in IDD models (Guan et al., 2026). Other proposed routes, including metabolite-driven regulation of intradiscal cell death, remain incompletely validated. However, the biological significance of this axis should not be interpreted as simple bacterial invasion of the disc. Rather, microbial-derived signals and host–microbe metabolic interactions appear to function as upstream regulators that reshape the inflammatory and metabolic landscape of a susceptible degenerative disc.

Disc and endplate studies implicate pyroptosis and ferroptosis in inflammatory and metabolic injury (Yang et al., 2021; Ma et al., 2022; Ma et al., 2024). Mitochondrial, redox, and iron-regulatory processes provide potential connections between these pathways (Zhang F. et al., 2025; Liang et al., 2026; Song et al., 2026; Wang W. et al., 2026), but their coordinated regulation by defined gut-derived metabolites remains to be demonstrated. Microbial metabolites may influence these processes by regulating inflammasome activation, antioxidant defense systems, macrophage polarization, mitochondrial quality control, and cellular metabolic adaptation. Nevertheless, direct evidence demonstrating that specific microbial metabolites reach the intervertebral disc at biologically relevant concentrations and causally regulate pyroptosis or ferroptosis remains limited. Current findings should therefore be regarded as mechanistically promising but requiring rigorous validation.

Therapeutically, targeting the gut–disc axis provides several potential opportunities, including microbiome remodeling, defined microbial consortia, bacterial extracellular vesicle-based therapy, metabolite supplementation or inhibition, mitochondrial restoration, regulated cell death modulation, and biomaterial-assisted targeted delivery. However, successful clinical translation will require overcoming several major barriers, including the heterogeneity of IDD phenotypes, uncertainty regarding microbial causality, limitations of current animal models, incomplete understanding of metabolite pharmacokinetics, and the absence of large-scale longitudinal clinical trials.

Future research should integrate microbiome sequencing, metabolomics, spatial transcriptomics, single-cell analysis, and functional genetic approaches to establish causal microbial–metabolic–cellular pathways. Ultimately, identifying patient-specific immune–metabolic endotypes and developing precision interventions targeting the gut–disc axis may provide a new therapeutic framework for disease modification rather than symptomatic management of intervertebral disc degeneration. 
